# Do We Still Need Chemistry for Water-Soluble Prodrugs? What Biocatalysis Already Does, and What It Does Not 

**DOI:** 10.3390/pharmaceutics18091166

**Published:** 2026-09-16

**Authors:** Federico Zappaterra, Domenico Meola, Francesco Presini, Lindomar Alberto Lerin, Pier Paolo Giovannini

**Affiliations:** Department of Chemical, Pharmaceutical and Agricultural Sciences, University of Ferrara, Via Luigi Borsari, 46, 44121 Ferrara, Italy

**Keywords:** prodrugs, water-soluble prodrugs, bioavailability, biocatalysis, lipase, glycosyltransferase, enzymatic esterification, chemo-enzymatic synthesis, green chemistry, green metrics

## Abstract

Poor aqueous solubility limits roughly 40% of marketed oral drugs and up to ~90% of development candidates. One established remedy is the prodrug strategy: the covalent attachment of a bioreversible promoiety that regenerates the parent drug in vivo. The same conjugation can move a molecule in either direction along the hydrophilic–lipophilic axis: lipophilization raises membrane and oil solubility for permeation-limited or topical uses, whereas hydrophilization raises aqueous solubility. This review addresses hydrophilization and asks whether enzymatic (biocatalytic) synthesis can replace conventional chemistry in building water-soluble prodrugs. We examine, class by class of hydrophilic promoiety (polyol and sugar esters, glycosides, amino acid esters, poly(ethylene glycol), and ionizable phosphates), the biocatalytic toolbox (lipases, acyltransferases, glycosidases, and glycosyltransferases), the molecular determinants of its selectivity, the pharmacokinetic consequences of hydrophilization, and the translation of these reactions to process, benchmarking each against the corresponding chemical route. A three-part test emerges: enzymes suffice, and frequently surpass chemistry, wherever an accessible hydroxyl or carboxyl must be functionalized regioselectively on a promoiety bearing no competing group and a moderate solubility gain is required. This is the regime of polyols, most sugars, and glycosides, with drug-conjugate aqueous-solubility increases of roughly 4-fold to 5500-fold; chemistry remains necessary for ionizable phosphates, amino acid esters, and PEG carriers. Two gaps limit translation: the field’s central ‘green’ claim is asserted far more often than it is measured (E-factor, PMI, life-cycle assessment), and in vivo pharmacokinetic data for enzymatically synthesized hydrophilizing prodrugs remain almost absent. We provide a decision flowchart that assigns each bond of a synthesis to biocatalysis or to chemistry, and reporting guidelines for the seven quantities that make a published E-factor recoverable.

## 1. Introduction

### 1.1. The Aqueous-Solubility Bottleneck in Drug Discovery and Development

Poor aqueous solubility has become the dominant physicochemical liability of the modern small-molecule pipeline. Contemporary medicinal chemistry, driven by combinatorial approaches and target-led design, tends to deliver candidates of higher molecular weight and greater lipophilicity, and with them a persistent solubility deficit: an estimated ~40% of marketed oral drugs and up to ~90% of the compounds in discovery pipelines are classified as poorly water-soluble [1,2,3]. Because dissolution in the gastrointestinal fluid is a prerequisite for absorption, low solubility translates directly into slow or incomplete dissolution, erratic and sub-therapeutic oral bioavailability and, ultimately, developmental attrition; physicochemical properties, lipophilicity foremost among them, are recognized contributors to candidate failure [4]. Solubility is therefore not a peripheral formulation detail but a gate-keeping property that decides whether an otherwise potent molecule can become a medicine.

The consequences of poor solubility are not uniform, however, and the Biopharmaceutics Classification System (BCS) makes the distinction precise. By ranking drugs along the two axes of aqueous solubility and intestinal permeability, the BCS separates compounds whose absorption is limited by dissolution (Class II: low solubility, high permeability) from those additionally limited by membrane transport (Class IV: low solubility, low permeability) [5]. The distinction is decisive for any solubilizing strategy: raising solubility rescues a Class II compound, whose only barrier is getting into solution, but leaves a Class IV compound still constrained by its poor permeability, a limit revisited in Section 5.2. A provisional BCS classification of the top-selling oral products indicates that about 40% of marketed drugs fall into the solubility-limited classes (BCS II and IV) [6]. The Developability Classification System (DCS) refines this picture for development by weighing solubility and permeability against dose and dissolution rate, and better predicts when solubility is genuinely the performance-limiting attribute [7]. The burden, moreover, is growing, and Lipinski’s analysis identifies the mechanism rather than the symptom. High-throughput screening reliably detects in vitro activity even in compounds of very poor thermodynamic solubility, and the readiest way to raise that activity is to add well-placed lipophilic groups, so the assay itself shapes the physicochemical profile of the hits it returns: leads from the HTS era carry higher molecular weight and log P, and lower solubility, than those that preceded it, and these are precisely the properties the rule of five flags as predictors of poor absorption or permeation [8]. About three-quarters of current development candidates now fall into BCS classes II and IV, a markedly higher proportion than among marketed drugs, so poor solubility is an increasingly upstream problem carrying a higher risk of attrition [9].

It also matters which solubility problem is being solved, because the two that prodrugs address are not the same. For a parenteral drug the goal is a concentrated, stable injectable, and the promoiety is chosen to make the molecule dissolve at a specific dose in a small volume; here, an ionizable group, most often a phosphate that endogenous alkaline phosphatases cleave once the drug is in the bloodstream, is the classic answer [10]. For an orally administered drug, the problem is dissolution-limited absorption, the defining case of BCS Class II, and raising solubility helps only so far as permeability allows, so a Class IV compound is not rescued by solubility alone [5]. The neutral enzymatic conjugates of Section 3 speak mostly to the oral problem; the ionizable prodrugs of Section 3.5 speak mostly to the parenteral one, a distinction the verdict map records for the phosphate class.

### 1.2. The Prodrug Strategy and the Solubility Axis: Lipophilization vs. Hydrophilization

A prodrug is an inactive, bioreversible derivative of a drug that regenerates the active parent in vivo through an enzymatic or chemical transformation and, among the strategies for poorly soluble molecules, it is one of the oldest and most clinically validated: roughly one in five small-molecule drugs approved between 2000 and 2008 was a prodrug, and more than 12% of the new small-molecule chemical entities approved in the following decade [10,11,12]. For solubility specifically, the approach works by covalently appending a promoiety, the solubilizing carrier group, that shifts the molecule along the hydrophilic–lipophilic axis [13]. Crucially, the same conjugation chemistry can move a drug in either direction: grafting a lipophilic group (lipophilization) raises membrane and oil solubility for topical or permeation-limited applications, whereas grafting a polar group (hydrophilization) raises aqueous solubility. Ketoprofen exemplifies the hydrophilizing route: its single carboxylic acid handle has been conjugated enzymatically to saccharides to give markedly more water-soluble derivatives [14]. The lipophilizing direction has been reviewed extensively elsewhere [15] and lies outside our scope; this review addresses the hydrophilizing direction, the enzymatic construction of water-soluble prodrugs.

The gain a promoiety delivers depends first on whether it carries a charge. A neutral, polar carrier such as a glycol, a sugar, or a short poly(ethylene glycol) chain typically increases solubility only two- to three-fold on its own, whereas an ionizable group such as a phosphate or an amine can raise it by several orders of magnitude [10]. The gains recorded for the neutral, enzymatically built conjugates of Section 3 (roughly 4-fold to 5500-fold, and, for the phytosterol esters, larger still) therefore seem at first to defy the rule. They are measured against parent drugs of vanishingly low baseline solubility, and the appended sugar or polyol does more than add polarity: it disrupts the crystal packing that held the parent insoluble in the first place [13]. The fold-change is large not because a neutral promoiety has become an ionizable one, but because two different barriers are being lowered. Dissolution requires both that a molecule leave its crystal and that the cavity it occupies in water be solvated, and Stella and Nti-Addae have separated the drugs limited by the first from those limited by the second: the high-melting, highly crystalline ‘brick dust’ molecule, whose crystal packing energy dominates, from the low-melting ‘grease ball’, whose difficulty is poor interaction with the solvent [13]. Adding a polar surface addresses the second barrier, which is the situation the two- to three-fold value describes, whereas disrupting the packing addresses the first, and is treated as a route to solubility distinct from attaching a polar or ionized promoiety [10]. The parents behind the largest gains of Table 3 are crystalline solids whose insolubility is most plausibly packing-limited, so the neutral-carrier results and the textbook expectation describe different mechanisms rather than contradicting one another. The reading remains inferential, however, since none of the syntheses surveyed reports the melting point, enthalpy of fusion, or solid-state characterization that would establish it.

**Figure 2:**
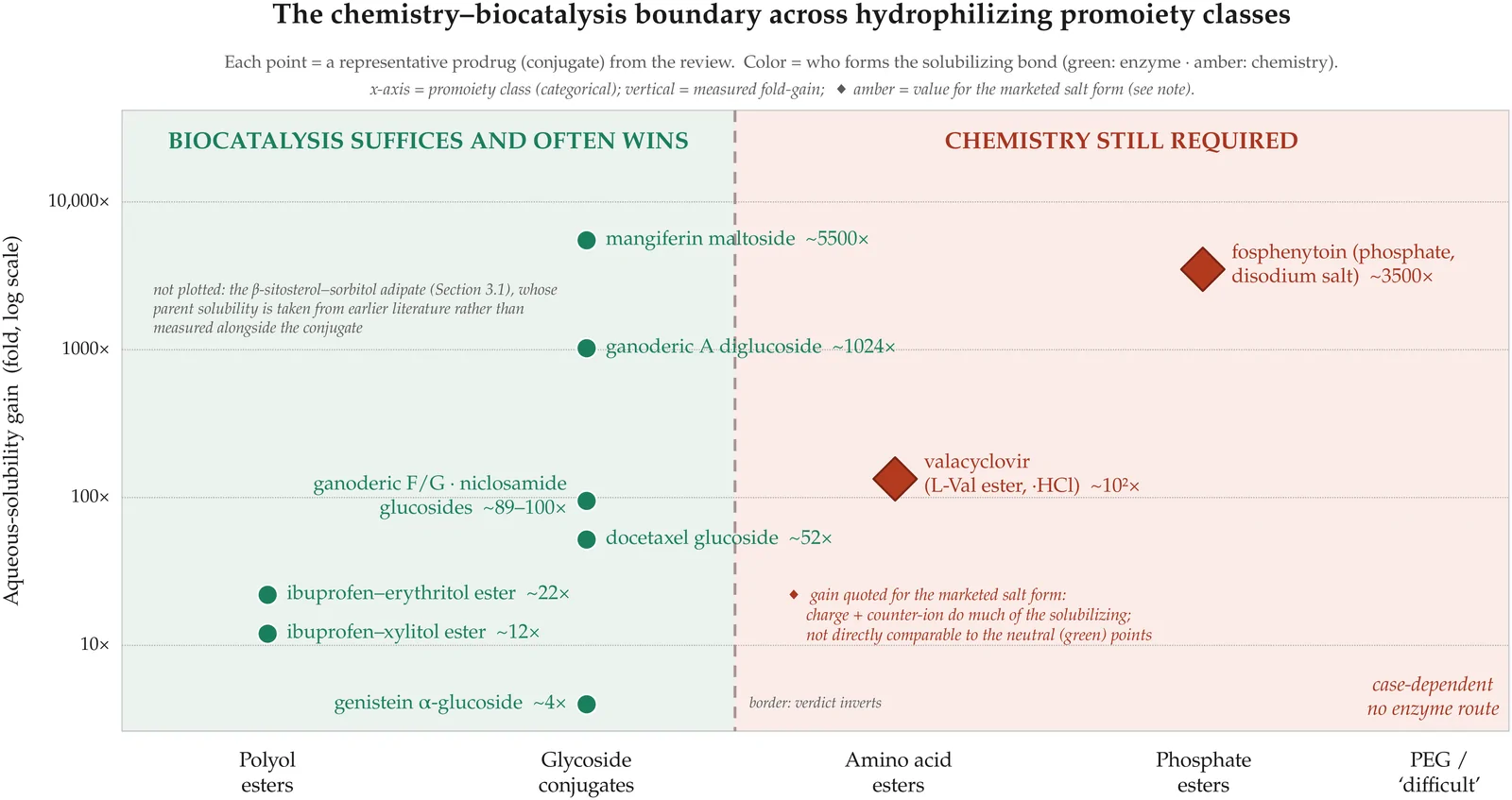
The chemistry–biocatalysis boundary across hydrophilizing promoiety classes. Each point is a representative prodrug of this review, placed by promoiety class (horizontal, categorical) and measured aqueous-solubility gain (vertical, log scale); color marks who forms the solubilizing bond, enzyme (green) or chemistry (amber); diamonds mark gains quoted for a marketed salt (fosphenytoin disodium, valacyclovir hydrochloride), where the charge and its counter-ion contribute much of the solubilization, so those values are not strictly comparable with the un-ionized green points. One point departs from that rule: docetaxel glucoside is chemo-enzymatic and is drawn green because a glycosidase forms the glycosidic bond of the sugar block, whereas the bond joining that block to the drug is made chemically (Section 3.2). It is the only such point plotted; the ketoprofen conjugates, enzymatic at the bond to the drug but chemical in the preparation of the acyl donor, are absent because no fold-gain is reported for them.

A second caveat is one of accounting rather than mechanism: the largest gains quoted anywhere in this review are not neutral conjugations but ionizations. The marketed phosphate and amino acid ester prodrugs are isolated and dosed as salts, fosphenytoin as its disodium salt (≈142 mg/mL, equivalent to ~88 mg/mL of phenytoin and a gain of roughly 3500-fold), and valacyclovir as its hydrochloride (≈174 mg/mL, about two orders over acyclovir), so much of their apparent solubility gain is contributed by the ionized group and its counter-ion, not by the covalent promoiety itself. Such salt-form values are not strictly comparable to the neutral, un-ionized conjugates that the enzymatic routes deliver, and the two are kept distinct throughout (in the text, in Table 3 and in Figure 2), the largest fold-changes sitting on the chemistry side precisely because they are realized as salts.

A third caveat mirrors the second and belongs beside it, because it runs the other way. Several of the drugs hydrophilized enzymatically in Section 3 are themselves carboxylic acids, and esterifying that acid to a polyol consumes the one group in the molecule that would have ionized. Ibuprofen and ketoprofen are weak acids, ketoprofen with a p*K*_a_ of 3.89 [7], so both are largely dissociated, and correspondingly far more soluble, at intestinal pH. The parent values against which their conjugates are compared, 21 mg/L for ibuprofen, are those of the un-ionized free acid in unbuffered water. That is the appropriate comparison for the stomach, where the acid is neutral and its dissolution genuinely limits absorption, and it is the deficit these prodrugs were designed to correct [16]; it is a generous one for the intestine, where the parent already dissolves as its anion. Both sets of values are therefore measured against favorable denominators, in opposite ways, and neither set of fold-changes should be read as a like-for-like ranking.

### 1.3. Chemistry vs. Biocatalysis for Building the Bond, and the Question This Review Asks

Once a hydrophilizing promoiety is chosen, the bond that attaches it can be forged by classical synthetic chemistry or by an enzyme, and the two routes differ in ways that matter for this review. Chemical acylation, glycosylation, or phosphorylation are general and robust, but on the polyhydroxylated promoieties central to hydrophilization they discriminate neither how many nor which hydroxyls react, so reaching a single monosubstituted product demands protecting-group manipulation, together with activated reagents and, often, forcing conditions. The two kinds of control are not equally important, and for a promoiety chosen purely to raise solubility they answer different questions. The degree of substitution governs the effect itself: every additional acylation both consumes a free hydroxyl and appends a second lipophilic drug unit, so a diester is more lipophilic than the corresponding monoester and moves the conjugate back along the axis it was meant to travel. The position of the ester barely changes the polarity, but it fixes the product as one chemical entity rather than a mixture of constitutional isomers, and it sets the steric environment of the bond and therefore the rate at which the drug is released (Section 5.3). Selectivity is thus wanted for two distinct reasons, the first for solubility and the second for identity and for control of reversion. Biocatalysis offers the opposite profile: hydrolases, glycosyltransferases, and related enzymes act with high regio- and stereoselectivity under mild, near-neutral conditions, frequently discriminating one hydroxyl among several without any protection and with a lower environmental burden [17,18]. These attributes align the enzymatic route with the principles of green chemistry (fewer steps, fewer auxiliaries, milder energy demand) that increasingly govern process choice in pharmaceutical synthesis [19,20,21]. Successive waves of protein engineering (enabled by DNA sequencing, gene synthesis, and directed evolution) have moved biocatalysis from a niche curiosity to a mainstream, industrially proven synthetic strategy now competitive with chemocatalysis and increasingly able to access even new-to-nature reactivity [22,23]. This contrast frames the question the title poses, and that the remainder of the review answers class by class: given that biocatalysis can build these bonds, do we still need chemistry for water-soluble prodrugs?

**Figure 1:**
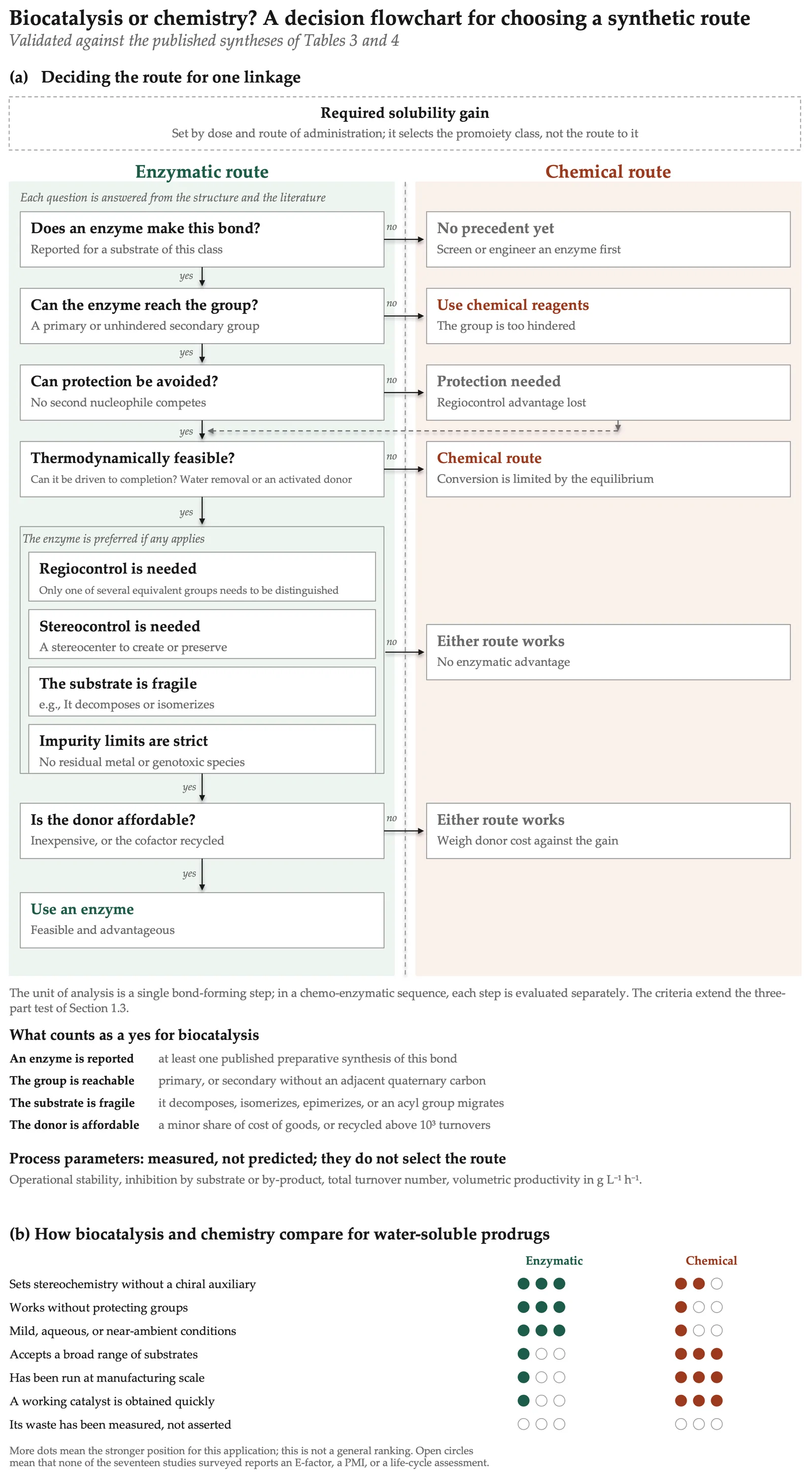
Biocatalysis or chemistry? A decision flowchart for choosing a synthetic route. (**a**) The chart is applied to one bond at a time, so that in a chemo-enzymatic sequence, each bond is assessed separately and may be assigned to a different route; what counts as a yes for each question is set out beneath the chart. (**b**) How biocatalysis and chemistry compare for water-soluble prodrugs; open circles mark a comparison that the surveyed literature has not established. The flowchart was validated against the twenty published syntheses collected in Table 3 and Table 4, the seventeen enzymatic routes of Table 3, and the three marketed chemical prodrugs of Table 4: fosphenytoin, valacyclovir. and valganciclovir.

A single criterion governs the class-by-class answers that follow. Biocatalysis is decisive wherever the synthetic difficulty is one of discrimination, both regarding which and how many hydroxyls react, and wherever the promoiety carries no second reactive group to compete for the catalyst; this is the regime of the polyols and sugars, where a lipase or glycosyltransferase delivers a single monosubstituted product in one step that the chemical route can reach only by protection and deprotection. Chemistry reasserts itself the moment that condition breaks: when the promoiety brings a competing nucleophile that must be masked in any case, as with the α-amine of an amino acid; when the solubilizing group is ionizable and demands a dedicated bond-forming chemistry of its own, as with a phosphate; or when the carrier is a featureless polymer that presents no competing sites for regioselectivity to distinguish, as with poly(ethylene glycol). The verdict map of Table 1 is, in essence, this criterion applied one class at a time [10]. To keep the comparison disciplined, we apply the same three-part test to every class that follows: an enzymatic route is expected to win when the drug or promoiety offers an accessible –OH or –COOH handle, when the promoiety carries no second reactive group that would compete for the catalyst, and when a neutral, moderate solubility gain suffices. These three conditions are the ones on which this review turns, and they are carried into Figure 1, a decision flowchart intended to support a reader who takes this review as a starting point for a new water-soluble prodrug and who must choose between an enzymatic and a chemical route.

A hydrophilizing prodrug must satisfy two conditions, not one, and only the first is a question of synthesis. The bond that carries the solubilizing group must be buildable, the concern of this review’s title, but it must also be cleavable in vivo, reverting cleanly to the active parent at or before its site of action; a conjugate that is beautifully made but does not release its drug, or that masks the very feature on which the drug’s action depends, is a solubilizer and not a prodrug [10]. The two conditions belong to different chemistries: the bond is most often formed by the routes debated here, enzymatic or classical, while it is almost always broken by an enzyme in the body, a hydrolase or phosphatase acting after administration. Section 5 takes up this second condition; the sections between it and here establish only that the bond can be made.

### 1.4. Scope, Boundaries, and Roadmap of This Review

The scope of this review is deliberately narrow along two axes. First, in synthetic route, we consider only the enzymatic (biocatalytic) construction of the solubilizing bond, treating classical chemistry as the benchmark against which the enzymatic route is judged rather than as a subject in its own right. Second, in direction, we consider only hydrophilization, the attachment of polar promoieties to raise the aqueous solubility of poorly soluble small molecules and bioactives. Three adjacent fields are therefore explicitly excluded. Lipophilization, the opposite movement along the same axis, is the subject of dedicated reviews and is not treated here [15]. Solubilization by formulation (cyclodextrin inclusion complexes [24], pharmaceutical cocrystals [25], nanocrystals [26], and amorphous solid dispersions [27]) raises apparent solubility without forming a bioreversible covalent bond, and therefore does not yield a prodrug; comprehensively surveyed elsewhere [2], these approaches fall outside our definition. Finally, conjugation aimed at targeting rather than solubility, such as antibody–drug conjugates, addresses a different problem and is likewise out of scope [28].

Within these boundaries, the review is organized as a single question answered per promoiety class. Existing reviews treat the enzymatic branches in isolation (the lipophilization of phenolics [15] or the glycosyltransferase-mediated glycodiversification of small molecules [29]), but, to our knowledge, none frames the comparison across every class of hydrophilizing promoiety, or centers the prodrug and its pharmacokinetic fate, as we do here: whether, for each class of hydrophilizing promoiety, enzymatic synthesis is sufficient to displace chemistry. A recent critical evaluation makes this comparison for glycosylation specifically [30], whereas the present review spans all hydrophilizing promoiety classes. Comprehensive reviews of prodrug design and of lipase-mediated sugar ester synthesis exist alongside these [10,31,32], but they treat the promoiety, the pharmacology, or the reaction in isolation rather than the choice between the two synthetic routes. Two contributions are therefore specific to the present review: the explicit three-part test of Section 1.3, stated in advance and applied to every promoiety class in the verdict of Section 7, so that the answer can be disagreed with on stated grounds, and the reporting audit of Section 6.4, which converts the field’s most repeated justification, and the endpoint that would vindicate it, into measured gaps. Section 2 surveys the biocatalytic toolbox; Section 3 assembles the per-class evidence; Section 4 and Section 5 examine the molecular determinants of selectivity and the pharmacokinetic consequences of hydrophilization; Section 6 addresses process translation and the still-unquantified question of the route’s ‘greenness’; Section 7 delivers the verdict class by class; and Section 8 sets out what does not yet work and what is moving. The answer is previewed in the verdict map of Table 1 and shown against the measured gains in Figure 2: enzymes suffice, and often win, for polyol, sugar, and glycoside conjugates, whereas chemistry remains necessary for phosphates, amino acid esters, and PEG, and the comparative sustainability that motivates much of the field remains asserted rather than measured.

**Table 1:** Verdict map: does chemistry stay necessary for water-soluble prodrugs? A per-class answer to the title question.

Promoiety/Linkage Class	Chemistry Still Needed?	Rationale→Section
Polyol and sugar esters (accessible –OH/–COOH)	NO; enzymes suffice. MIXED where the acyl donor is prepared chemically from the drug	Regioselective, protecting-group-free, mild; core evidence→Section 3.1; MIXED for the ketoprofen–saccharide conjugates, enzymatic at the bond to the drug but starting from a vinyl ester prepared with mercury(II) acetate and sulfuric acid (Section 3.2)
Glycoside conjugates (GT/transglycosidase)	NO/Mostly enzymatic. MIXED where the drug offers no accessible hydroxyl	GTs glycosylate poorly soluble scaffolds directly (~4–5500×)→Section 3.2; bond formation only; reversion/retained activity is class-variable (→Section 5.2); MIXED for the docetaxel conjugate, where the enzyme builds the glycosyl block and EDCI with DMAP forms the bond to the drug, under benzyl and triethylsilyl protection (Section 3.2)
Phosphate esters	YES, chemistry dominates	Biggest solubility jumps, but realized as salts (parenteral/IV); kinases are ATP-dependent and, one engineered exception aside, narrow in scope; acid phosphatases operate at mM-to-M acceptor→Section 3.5
Amino acid esters	YES, largely chemical	Free amine competes; stereochemistry; enzymatic route hard→Section 3.3; one enzymatic attempt on record, limited by polymer from the free amine (Section 3.3)
PEG/‘difficult’ substrates	YES; chemistry in every reported case	Hindered tertiary alcohol on the drug side; no enzymatic route to the solubilizing bond→Section 3.4/Section 8.1/Section 8.2
Enzyme-vs-chemistry ‘greenness’	UNPROVEN	Claimed but rarely measured (E-factor/PMI/LCA): the gap→Section 6.4

## 2. The Biocatalytic Toolbox for Hydrophilizing Conjugation

**Scheme 1:**
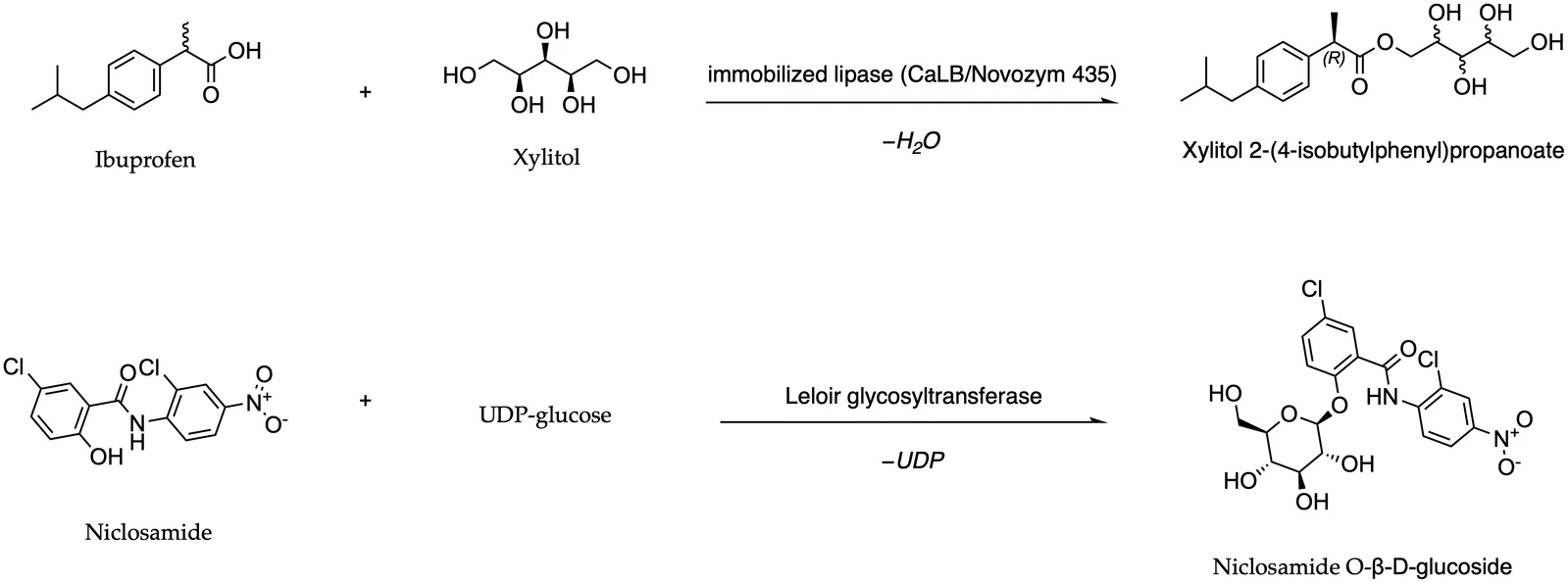
Biocatalytic hydrophilization of a poorly water-soluble drug, shown for the two bond types treated in this review. **Top**: An immobilized lipase (*Candida antarctica* lipase B, Novozym 435) esterifies the carboxylic acid of ibuprofen with the primary hydroxyl of xylitol, releasing water, to give xylitol 2-(4-isobutylphenyl)propanoate. **Bottom**: A Leloir glycosyltransferase transfers glucose from UDP-glucose onto the phenolic hydroxyl of niclosamide, releasing UDP, to give niclosamide 2-*O*-β-d-glucoside. Immobilization allows the biocatalyst to be recovered and reused: Novozym 435 gave 68 ± 3% conversion after five cycles, against 80% in the first, for the ibuprofen–xylitol ester [60], and a co-immobilized glycosyltransferase–sucrose-synthase cascade retained about 40% of its activity over fifteen cycles [61]; the two values are not directly comparable. A wavy bond marks a center whose configuration is not defined in the cited work, here the esterified polyol carbon; the ibuprofen center is drawn (*R*), the enantiomer CaLB is reported to prefer.

**Figure 3:**
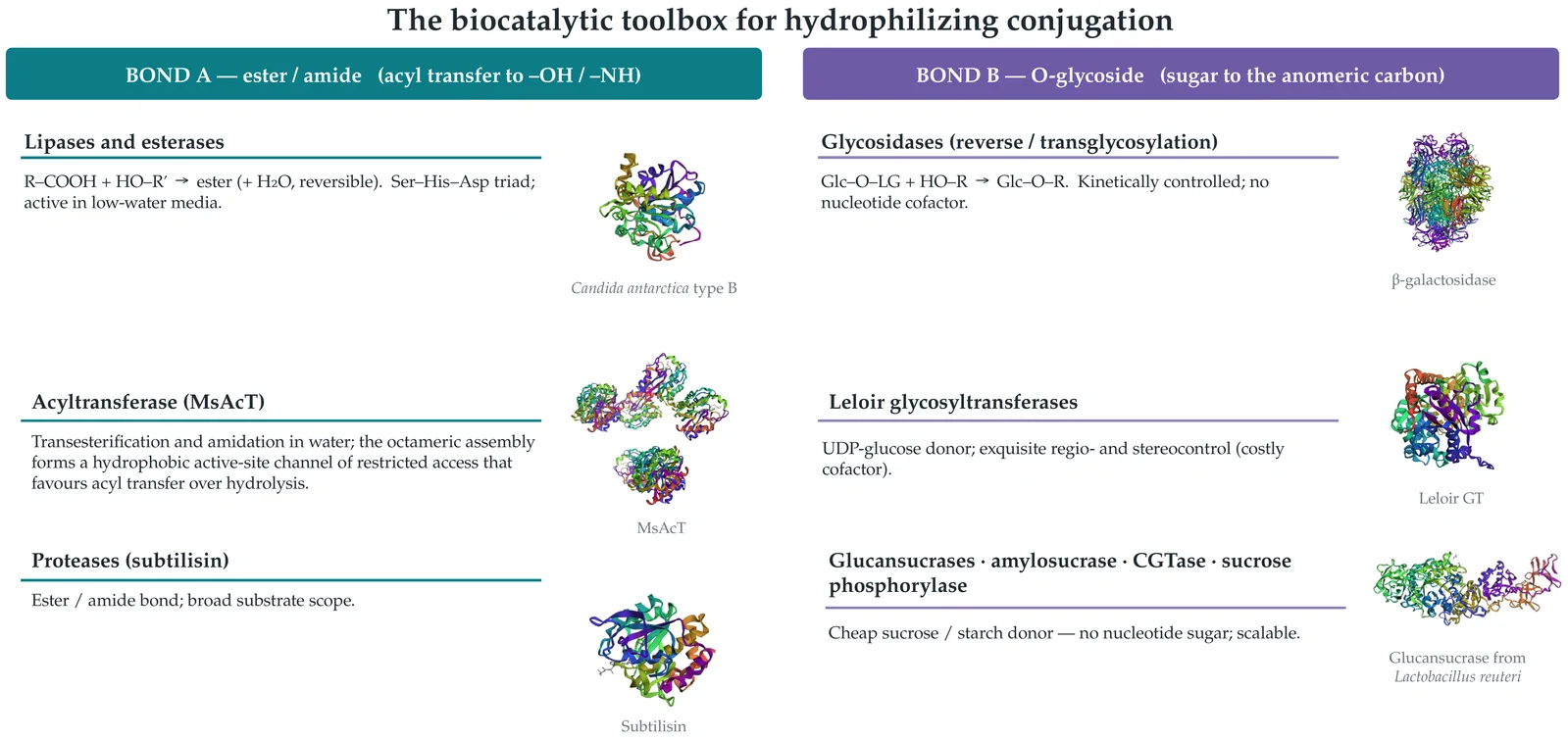
The biocatalytic toolbox for hydrophilizing conjugation. Enzyme families are organized into two mechanistic groups, the ester- and amide-forming hydrolases and acyltransferases (Section 2.1) and the *O*-glycoside-forming glycosidases and glycosyltransferases (Section 2.2), each annotated with the donor it requires and the conditions it imposes. Ribbon structures are taken from the Protein Data Bank (*Candida antarctica* lipase B, 1TCA; acyltransferase MsAcT, 2Q0Q; subtilisin, 1SCN; β-galactosidase, 1DP0; Leloir glycosyltransferase, 2C1Z; glucansucrase GTF180 from *Lactobacillus reuteri*, 3KLK); they illustrate the fold of enzymes in common use and are not, except where a study happens to have used the same protein, the specific catalysts of the syntheses reviewed in Section 3.

The instruments are set out here (Figure 3, Scheme 1), and set out comparatively, because every bond-forming enzymatic tool in this section has a classical chemical counterpart against which it will be judged. Where the enzyme is a lipase (or other hydrolase) or an acyltransferase, the chemical alternative is carbodiimide (Steglich) coupling, an acyl chloride, or direct acid-catalyzed (Fischer) esterification; where it is a glycosyltransferase or glycosidase, the alternative is chemical glycosylation (Koenigs–Knorr, Fischer glycosidation); where a phosphate must be introduced, the alternative is protecting-group phosphorylation. Those chemical routes are examined head-to-head in Section 6.3; here we set out the biocatalytic toolbox whose scope and limits determine, class by class, whether the enzyme can displace them.

### 2.1. Hydrolases, Acyltransferases, and Whole-Cell Systems

The workhorses of hydrophilizing conjugation are the hydrolases, and above all the lipases. These serine hydrolases operate through the canonical Ser–His–Asp catalytic triad and, unusually, retain and even gain activity in low-water organic media, where the hydrolytic equilibrium is driven back toward synthesis and, where substrate, stereo-, and regioselectivity can be tuned, sometimes inverted, by the solvent [33]. The lipase B from *Candida antarctica* (CaLB), sold in immobilized form as Novozym 435, is the field’s default catalyst: it acts on a broad range of primary alcohols and carboxylic acids and is markedly thermostable, while its A-type counterpart (CaLA) extends the reach of these yeast lipases even to sterically hindered secondary and tertiary alcohols [34]. This blend of promiscuity toward polyols and alcohols, mild operation, and commercial availability is precisely what makes lipases the natural tool for grafting a hydrophilic promoiety onto a drug bearing a –COOH or –OH handle, as the extensive record of lipase-made active pharmaceutical ingredients attests [20]; proteases and esterases extend the same logic to substrates and regiochemistries that lipases address poorly.

Two developments widen the toolbox beyond the classical lipase. First, the acyltransferase from *Mycobacterium smegmatis* (MsAcT) performs transesterification, amidation, and perhydrolysis directly in water: its octameric assembly forms a hydrophobic active-site channel of restricted access that favors acyl transfer over hydrolysis even in aqueous medium (its perhydrolysis-to-hydrolysis ratio, measured with hydrogen peroxide as the acceptor, is some fifty-fold higher than that of the best lipase tested), so that esters can be built without the anhydrous conditions hydrolases normally demand [35,36]. Making esters in water rather than by displacing an equilibrium in organic solvent is a genuine frontier for hydrophilic conjugation, where drug and promoiety are both water-soluble. Second, whole-cell biocatalysis uses the intact microbial cell as a self-contained package of enzymes and cofactors: it avoids enzyme purification, stabilizes the catalyst within its native envelope, and regenerates costly cofactors in situ, an advantage that becomes decisive for the cofactor-dependent glycosyltransferases considered next, and one already exploited to make flavonoid glucosides in engineered *Saccharomyces cerevisiae* once its endogenous glucosidases are deleted to stop them hydrolyzing the product [37,38]. CaLB is an extracellular enzyme, carrying a signal peptide at residues 1 to 18 (UniProt P41365), whereas for MsAcT, neither a signal peptide nor a subcellular localization is annotated (UniProt A0R5U7) and the enzyme can be purified from the soluble fraction of disrupted cells, which is consistent with an intracellular enzyme. Localization alone does not decide whether whole cells are the better format, since a secreted enzyme can be used in the culture broth without being isolated. What makes them useful is the cofactor and its regeneration: a lipase or an acyltransferase needs none, whereas a Leloir glycosyltransferase consumes a sugar nucleotide that only the intact cell regenerates.

### 2.2. Glycosidases and Glycosyltransferases: (Trans)glycosylation and Cofactor Economics

Sugars can be attached by an entirely different enzymatic logic, and the choice of enzyme is really a choice of sugar donor. Glycoside hydrolases can be run in reverse: under transglycosylation conditions, enzymes such as β-galactosidases and β-xylosidases transfer a sugar to an acceptor rather than hydrolyzing it, and have been used to assemble glycosyl blocks and glycosides of poorly soluble scaffolds [39]. The β-galactosidases include the enzyme marketed as lactase, the same activity (EC 3.2.1.23) under a trade name rather than a separate class, so where Section 3.2 sets two of them side by side, the difference lies in the product rather than in the enzyme: the *Kluyveromyces lactis* enzyme was used there to make a glucoside, the *Aspergillus oryzae* β-galactosidase to make a galactoside. Glycosyltransferases (GTs) achieve the same attachment with far higher regio- and stereocontrol: adopting one of two conserved folds (GT-A, metal-dependent; GT-B, metal-independent) and acting by an inverting or a retaining mechanism that fixes the α or β anomer, they deliver the sugar to a defined position and configuration [40]. Direct glucosylation of poorly soluble bioactives by GTs and maltogenic amylases has produced large aqueous-solubility gains, from mangiferin to niclosamide [41,42]. Two further sucrose- or starch-based transglucosidases broaden this toolkit: amylosucrase transfers glucose from sucrose to acceptors such as resveratrol, whose α-glucoside is 13.5-fold more water-soluble than the aglycone [43], while cyclodextrin glucanotransferase (CGTase) transglucosylates poorly soluble flavonoids directly in water, raising the aqueous solubility of baicalin roughly 190- to 320-fold as its mono- and diglucosides [44].

The practical divide within this branch is economic. Leloir GTs require an activated nucleotide sugar such as uridine diphosphate glucose (UDP-glucose), too costly to consume stoichiometrically unless regenerated in situ, typically by coupling the reaction to sucrose synthase, which recycles UDP back to UDP-glucose at the expense of cheap sucrose [45]. Non-Leloir enzymes avoid the problem altogether: family GH70 glucansucrases and transglucosidases transfer glucose directly from sucrose (and related amylases from starch), so that no nucleotide cofactor is needed at all [46]. It is largely this donor economy, rather than any difference in the glycosidic bond itself, that decides which glycosylation route is practical at scale, an argument revisited when process costs are weighed in Section 6.

### 2.3. Engineering the Catalyst: Directed Evolution, Glycorandomization, Promiscuity Expansion

Where a natural enzyme lacks the required scope, it can be engineered to acquire it. Directed evolution, iterative rounds of mutagenesis and screening, has become routine and, since its recognition with the 2018 Nobel Prize in Chemistry, an emblematic means of tailoring biocatalysts to non-natural substrates and conditions [47]. For the glycosyltransferases central to Section 2.2, evolution has broadened acceptor promiscuity, the basis of glycorandomization, and has converted carbohydrate-processing enzymes into synthetically useful transferases [48,49]. The reach of the approach is captured by multi-enzyme design at manufacturing scale: the synthesis of the antiviral islatravir was compressed into a three-step, nine-enzyme in vitro cascade in which five enzymes were evolved to act on non-natural substrates, showing how far engineered biocatalysis has moved from single transformations toward complete synthetic routes [50]. The same rational-design logic underpins the molecular determinants of selectivity treated in Section 4.

### 2.4. Reaction Media, Acyl-Donor Strategy, and Thermodynamic Control

Because hydrophilization joins a polar promoiety to an often apolar drug and releases water, the reaction medium is a control variable rather than a passive backdrop. Working in low-water organic solvent shifts a hydrolase toward synthesis and can sharpen or even invert its selectivity [33]. Solvent choice is now also a sustainability decision: the CHEM21 guide scores solvents on safety, health, and environment and classifies them, on a separate scale, by the fraction of carbon that is bio-based, although it ranks many bio-derived solvents as problematic, because their high boiling points make recovery difficult [51]. Deep eutectic solvents fall outside that guide and have been assessed separately for lipase catalysis [52]. One synthesis in this survey shows what that choice is worth in practice: xylitol monoferulate is made in *tert*-amyl alcohol, a solvent that holds both a hydrophilic polyol and a sparingly soluble phenolic acid in a single phase [53], building on the broader methodology of lipase-catalyzed sugar ester synthesis in non-aqueous media [32].

The second control variable is the position of the equilibrium. An esterification is inherently reversible, and two strategies drive it toward product formation. The first is thermodynamic: water is a co-product, so removing it, with molecular sieves or under vacuum, lowers the thermodynamic water activity *a*_w_ and shifts the position of the equilibrium toward the ester. Water activity is not the same quantity as water content, since the same amount of water is held more tightly by a polar solvent or a hydrophilic support than by an apolar one, and it is the activity that the equilibrium and the enzyme both respond to. The second strategy is kinetic, and does not move the equilibrium but removes it by using an activated acyl donor. Vinyl and isopropenyl esters are the classic activated donors: the released enol tautomerizes irreversibly (vinyl esters to acetaldehyde, isopropenyl esters to acetone), so the reaction cannot run backwards. This acyl-donor strategy was first demonstrated for the lipase synthesis of monoacylglycerols [54]. The convenience carries a cost: the liberated acetaldehyde is reactive and can deactivate the enzyme through covalent modification, so the gain in conversion must be balanced against biocatalyst stability [55]. Controlling water activity and the synthesis-versus-hydrolysis balance is thus central to any preparative hydrophilization.

### 2.5. Immobilization, Operational Stability, and Reuse

Whatever the enzyme and medium, practical and commercial viability usually requires that the biocatalyst be immobilized. Fixing the enzyme to a support (by adsorption, encapsulation, or carrier-free cross-linking) improves operational stability and, above all, enables recovery and reuse across many cycles while lowering the risk of protein residues in the product [56]. Immobilization is more than a packaging step: confinement on a support can itself modify activity, specificity, and selectivity, sometimes detrimentally but sometimes favorably, as in the interfacial activation of lipases on hydrophobic carriers [57]. Novozym 435, CaLB adsorbed on a macroporous acrylic resin, is the archetype, and its performance, stability, and limitations have been analyzed in detail [58]. Direct spectroscopic evidence supports this: infrared microspectroscopy of an immobilized ω-transaminase showed that the carrier’s surface chemistry reshapes the enzyme’s secondary structure and that a reaction water activity above 0.90 markedly improves turnover, tying support choice and reaction medium directly to catalytic performance [59]. Read beside the equilibrium argument of Section 2.4, this is a limit rather than a contradiction: transamination releases no water, so there the activity acts only on the hydration of the protein, whereas for a hydrolase, the same variable must be set low enough to drive synthesis and high enough to keep the enzyme mobile. Immobilization also provides the physical form required for the packed-bed, continuous-flow operation that Section 6 takes up as the bridge to process.

## 3. Hydrophilizing Promoieties and the Prodrugs Made from Them

### 3.1. Polyols and Sugar Alcohols (Xylitol, Sorbitol, Erythritol, Glycerol, Mannitol)

Biocatalysis is applied most often to a reaction that appears simple: grafting a polyol onto a poorly soluble drug through a single ester bond. A polyol such as xylitol or sorbitol presents five or six hydroxyls, of which only two are primary; the classical chemical route to a single defined monoester must therefore protect all but one, couple, and deprotect, spending steps and reagents to buy a regiochemistry that a lipase delivers in a single operation. An enzyme is favored here not by the bond itself but by the selectivity with which it must be formed.

One series carries the same drug, ibuprofen, toward the hydrophilic end of the axis while the biocatalyst and the reaction medium are progressively simplified. Ibuprofen is an apt test case: an aqueous solubility of only ~21 mg/L renders its oral absorption dissolution-limited [16], precisely the deficit a hydrophilic promoiety is meant to correct. In a first iteration, ibuprofen was esterified directly with sorbitol in a biphasic hexane/water system using free porcine pancreatic lipase (a deliberately inexpensive, non-immobilized catalyst), with the water content itself governing the esterification/hydrolysis balance [16]. A second iteration replaced both the biphasic system and the free enzyme with immobilized *Candida antarctica* lipase B (Novozym 435) in a single organic phase: simpler to operate, regioselective for the primary hydroxyl of xylitol, and giving an ester twelve times more soluble in water than ibuprofen, on a determination whose method is not stated [60]. A third iteration dispensed with added solvent altogether: (*S*)-ibuprofen was condensed with glycerol under solvent-free conditions catalyzed by immobilized *Rhizomucor miehei* lipase, the glycerol serving at once as substrate and as enzyme stabilizer [62]. The ibuprofen–erythritol ester reaches 463 µg/mL by the turbidimetric method, twenty-two times the solubility of ibuprofen itself [62], and the glyceric ester of ursodeoxycholic acid reaches 123 mg/L against 20 mg/L for the parent bile acid, a six-fold gain; the ester formed at the C-24 carboxyl, with NMR placing the glycerol attachment on a primary hydroxyl [63]. Read in sequence, these three iterations show that the enzymatic route is not a fixed protocol: catalyst cost, phase behavior, and solvent burden were each reduced without loss of selectivity. Beyond ibuprofen, the same protecting-group-free, CaLB-catalyzed strategy has been applied across a range of poorly soluble bioactives and polyols: nine cinnamic-acid derivatives were esterified with the polyol erythritol, with molecular docking rationalizing how aromatic-ring substituents govern conversion [64], and the lipophilic preservative sorbic acid was converted to a more hydrophilic glycerol ester by the same lipase [65]. Reaction media and conversions for the whole series are collated in Table 3.

That selectivity is not accidental. Molecular docking of the two ibuprofen enantiomers predicts opposite enantiopreferences for the two lipases, CaLB favoring the (*R*)-acid, the *Rhizomucor miehei* enzyme the (*S*), through the near-attack geometries treated in full in Section 4 [62].

A further refinement turns the promoiety from a passive solubilizer into a second active principle. When the acyl partner is itself bioactive, the conjugate becomes bifunctional: esterification of ferulic acid, an antioxidant, with xylitol yields xylitol monoferulate, a molecule in which both halves carry activity [53]. Here the enzyme also resolves a polarity mismatch that would frustrate a one-pot chemical reaction, since a single green solvent (*tert*-amyl alcohol) dissolves both the sparingly soluble phenolic acid and the strongly hydrophilic polyol, with water withdrawn by molecular sieves to drive the equilibrium [53].

The approach is not limited to substrates that carry a carboxylic acid. Phytosterols, which offer only hydroxyl handles and so cannot be esterified to a polyol directly, have been joined through a divinyl adipate linker in a fully enzymatic two-step sequence [66]. Divinyl adipate is the bis(vinyl ester) of adipic acid, and the bridge is built one ester at a time: a *Candida rugosa* lipase acylates the sterol 3β-hydroxyl with the first, releasing acetaldehyde and leaving the second intact, and an alkaline protease from *Bacillus subtilis* then transfers that second ester to a hydroxyl of sorbitol. The product is a 1:1:1 sterol–adipoyl–polyol diester, and which hydroxyl of the polyol is acylated is not established. Aqueous solubility rises to 7.89 mM at 35 °C, against the value of 2.8 × 10^−6^ mM reported for the parent phytosterols, and bioaccessibility rises in parallel in vitro. The polyol step runs in pyridine, which the authors identify as a residue risk in need of replacement. The same logic carries to bioactives that are not drugs: immobilized *Candida antarctica* lipase B transesterifies the terminal methyl ester of the food colorant bixin with sorbitol, giving the sorbitol ester of norbixin; the improved hydrophilicity is anticipated by the authors rather than measured [67]. Two features of this sequence bear on Section 6: the activated vinyl donor that renders the first step irreversible liberates acetaldehyde, and the pyridine of the second step is a solvent whose toxicity is difficult to reconcile with a claim of greenness.

Taken together, the polyol studies are the class for which the verdict map is most firmly enzymatic. For this class, the answer to the title’s question is not that enzymes will do but that enzymes are the better tool, because the regiochemistry they deliver in a single step is exactly what the chemical route spends protection and deprotection to obtain. This is the first of the negative answers recorded in Table 1, and the one that rests on the largest number of studies.

### 3.2. Mono- and Disaccharides: Ester- and Glycoside-Linked Conjugates

If polyols make the cleanest case for enzymatic hydrophilization, mono- and disaccharides make the richest one, for a sugar can be attached to a drug by two mechanistically distinct routes: an ester bond, forged by the very hydrolases already met, or a glycosidic bond, forged by an entirely different class of enzyme.

The ester route extends the logic of Section 3.1 from polyols to sugars, and it accommodates two further demands: a poorly soluble, high-value drug and, where that drug is chiral, a single enantiomer. Docetaxel illustrates both demands. Because the taxane offers no convenient handle for direct sugar esterification, the conjugate was assembled chemo-enzymatically: a β-galactosidase or a β-xylosidase first built a carboxyethyl β-d-glycoside by transglycosylation, and this pre-formed glycosyl block was then coupled chemically to the C-7 hydroxyl of docetaxel, through a sequence that benzylated the sugar hydroxyls, silylated the drug’s C-2′ hydroxyl, joined the two with EDCI and DMAP, and removed both protecting groups by hydrogenolysis [39]. The glucoside was 52-fold more water-soluble than docetaxel and, with the xyloside, was hydrolyzed back to the drug by cancer-cell enzymes, whereas the galactoside was converted to only 45% against 88 and 76% for the other two, and was the least cytotoxic of the three—an early signal, revisited in Section 5, that the sugar governs not only solubility but release. Two features of the sequence qualify the result: the enzyme forms the glycosidic bond, but the bond to the drug is made chemically and carries the full protecting-group burden that the polyol esters of Section 3.1 avoid, and the enzymatic step is here the least productive of the sequence, the three glycosylations returning 22 to 35% against 85 to 95% for each of the chemical steps that followed.

The ketoprofen–saccharide conjugates make the stronger claim, because both steps are enzymatic. A lipase from *Mucor miehei* first resolves the racemic ketoprofen vinyl ester by hydrolyzing the (*R*)-ester, leaving the pharmacologically active (*S*)-vinyl ester in 90% enantiomeric excess, and a protease from *Bacillus licheniformis* then transesterifies it onto a panel of six mono- and disaccharides and one sugar alcohol, regioselectively at a primary hydroxyl in every case. The yields are uneven: 87% for the mannoside, but 23 to 50% for four of the seven conjugates, the disaccharides faring worst [14]. Every conjugate was more hydrophilic than the parent drug, as measured by its partition coefficient, and hydrolysis at pH 7.4 returned 37% of the ketoprofen from the glucoside and 47% from the maltoside within 24 h, and close to 90% over seven days. Three features recur from Section 3.1 and matter for Section 6: the reaction is driven irreversibly by a vinyl ester, again at the cost of liberating acetaldehyde; it is run in a 1:1 pyridine/*tert*-butanol mixture, chosen because unprotected sugars dissolve in little else, so that the solvent burden of this route is set by the promoiety rather than by the enzyme; and the racemic vinyl ester on which both enzymatic steps depend is itself prepared with mercury(II) acetate and sulfuric acid in refluxing vinyl acetate, the same class of heavy-metal promoter for which the classical Koenigs–Knorr glycosylation is faulted in Section 6.3.

The glycosidic route dispenses with the ester entirely and attaches the sugar through its anomeric carbon, but it forces a choice of enzyme that is really a choice of donor economy. Leloir glycosyltransferases transfer sugars with exquisite regio- and stereocontrol, yet demand an activated nucleotide sugar such as UDP-glucose, a reagent too costly to consume stoichiometrically at scale unless it is regenerated. Glucansucrases and transglycosylating amylases sidestep this by transferring glucose from cheap, abundant donors, like sucrose or starch, and it is largely this economic distinction, rather than any difference in the bond itself, that decides which glycoside route is practical.

The trade-off is visible across three representative systems. Engineering the glucansucrase GTF-D from *Streptococcus mutans* expanded its acceptor scope so that it glucosylates flavonoids (catechin, genistein, daidzein, silybin) from sucrose rather than a nucleotide sugar, raising the solubility of genistein roughly four-fold; notably, the products are α-glucosides, whereas the glycosides that occur in nature are β, so the enzyme yields compounds new to the natural-product reservoir [49]. A maltogenic amylase from *Parageobacillus galactosidasius* glycosylates the xanthone mangiferin from maltodextrin, and the maltosyl-α-(1→6) product is some 5500-fold more soluble than mangiferin while retaining its antioxidant activity, a solubility gain obtained with no nucleotide cofactor at all [41]. Where the nucleotide-sugar cost is accepted, the structural reward can be large: sequential action of two *Bacillus* glycosyltransferases on ganoderic acid A, one at the C-15 hydroxyl and the other at the C-26 carboxyl, furnished a diglucoside 1024-fold more soluble than the parent triterpenoid [68].

The same *Bacillus* enzymes extend the pattern to related triterpenoids, and show that the position matters as much as the sugar: BsGT110 rendered ganoderic acid F 89-fold and the 26-*O*-glucoside of ganoderic acid G 97-fold more soluble than their aglycones, while BsUGT489 glucosylating the same acid at C-3 gave only a 54-fold gain [69,70].

A solubility gain does not by itself make a prodrug, and one result in this class separates the two directly. Enzymatic *O*-glucosylation of niclosamide, achieved with engineered plant glycosyltransferases, made the drug 100-fold more soluble at physiological pH, and simultaneously abolished its antimicrobial and antiviral activity [42]. The glycoside is therefore useful only as a true prodrug, one that must shed its sugar at the target site to act; the solubility–permeability tension is taken up in full in Section 5. In the verdict map, the sugars therefore fall into two groups. Where the bond is glycosidic, the enzyme does the whole job and the class behaves like the polyols before it. Where the bond is an ester, chemistry re-enters: it made the drug–block linkage for docetaxel and the racemic vinyl ester on which the ketoprofen route depends. These two syntheses are therefore chemo-enzymatic rather than enzymatic, and Table 1 and the caption of Figure 2 record them as mixed. They are mixed at different points, and the difference decides how much biocatalysis has replaced: for docetaxel, the chemistry forms the bond to the drug itself, whereas for ketoprofen, it only prepares the acyl donor that the enzyme then uses. Both classes are collated in Table 3, and the open question is no longer whether the bond can be formed enzymatically, but whether the conjugate reverts to an active drug. With the next class, the amino acids, the answer to the title’s question changes outright.

### 3.3. Amino Acids and Small Peptides

**Scheme 2:**
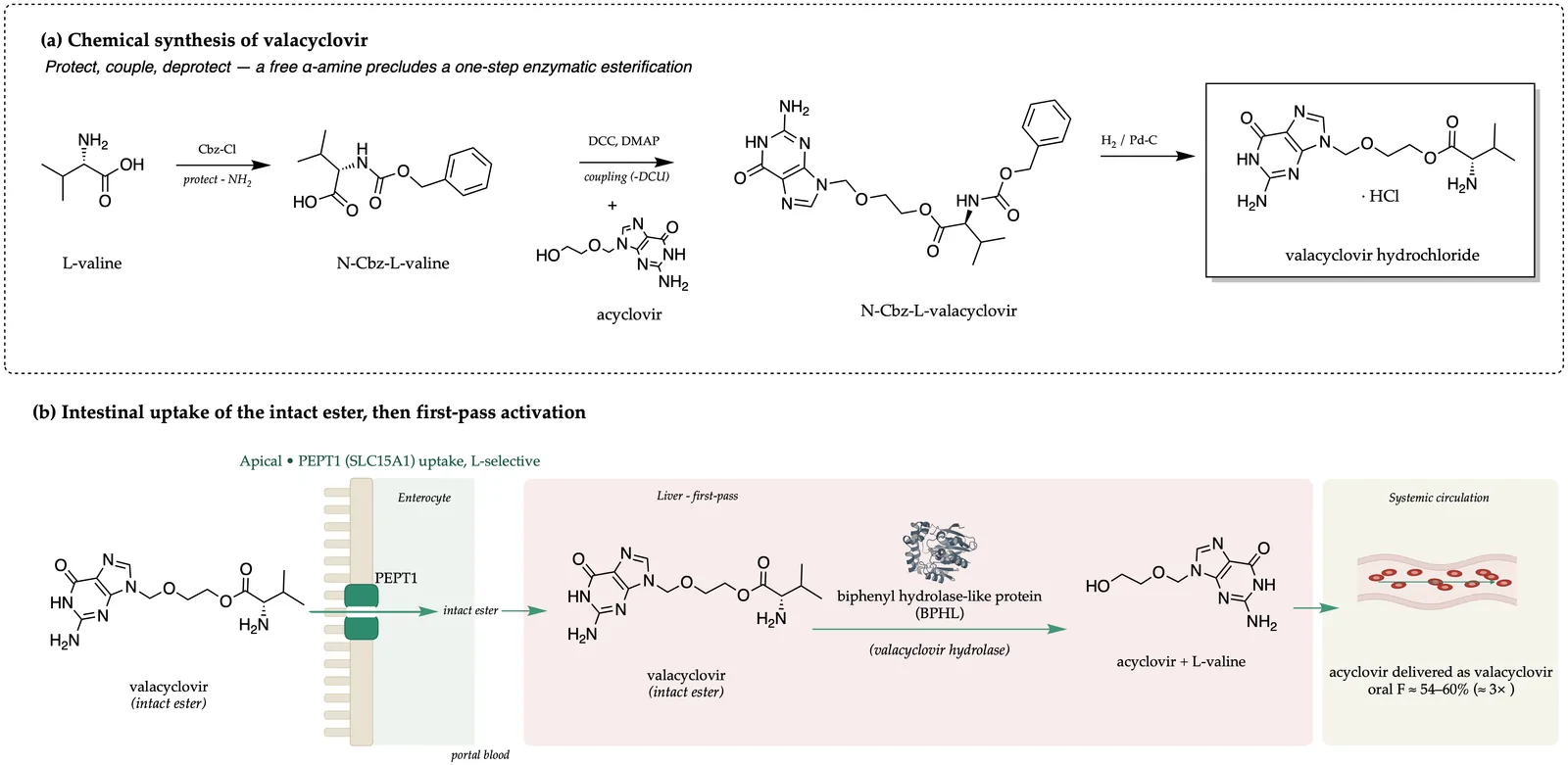
Amino acid ester prodrugs are transporter-targeting rather than merely solubilizing, shown for valacyclovir, the l-valyl ester of acyclovir. (**a**) Protect–couple–deprotect chemistry: the α-amine of l-valine is masked (Cbz-Cl), the acid is coupled to the acyclovir hydroxyl (DCC, DMAP, with dicyclohexylurea as the stoichiometric co-product), and the mask is removed by hydrogenolysis in the presence of HCl, which delivers the drug as its hydrochloride [72]. (**b**) In vivo, PEPT1 (SLC15A1) drives stereoselective active uptake of the intact ester across the enterocyte apical membrane [73], which is then hydrolyzed presystemically in the liver by biphenyl hydrolase-like protein (BPHL, PDB 2OCG) to acyclovir and l-valine [74].

The amino acids are the first class in which the answer to the title’s question moves toward chemistry, and it moves without settling there, because the obstacle is one of selectivity rather than of principle. An amino acid raises aqueous solubility by the same charge-driven logic that governs the phosphates of Section 3.5, though far more gently: its α-amino group, protonated at gastric and physiological pH, supplies the ionizable handle. Acyclovir is the standard example. The antiherpetic is sparingly soluble, 1.2 to 1.6 mg/mL at room temperature [71], and erratically absorbed, with an oral bioavailability of only 15–21%; esterification of its hydroxyl with l-valine gives valacyclovir (Scheme 2), soluble as its hydrochloride at about 174 mg/mL, a rise of roughly two orders of magnitude, though carried largely by the ionizable α-amino group as its hydrochloride salt rather than by the valyl ester itself (whose primary role is PEPT1-mediated uptake), and far better absorbed by mouth [72].

The bond is made by chemistry rather than by the enzyme, and the reason lies in the promoiety itself. The amino acid carries, beside the carboxyl that must become the ester, a free α-amine that is an equally willing nucleophile; unprotected during a chemical coupling, it condenses to peptides or consumes the activating reagent, and its stereocenter is fragile. The classical route therefore masks the amine as an *N*-Cbz or Boc carbamate, couples with a carbodiimide, and strips the mask by hydrogenolysis or acid (the protection a lipase avoids in the polyol series), while the strongly basic coupling catalyst still drives partial racemization: 2 to 3% of the d-isomer was measured in most batches of the *N*-Cbz-valyl intermediate and attributed by the authors to DMAP [72]. Stereochemistry matters because absorption is itself stereoselective, the l-esters being taken up markedly better than their d-counterparts and the racemates falling in between [72], a preference later assigned to the intestinal peptide transporter PEPT1 [73]. The marketed amino acid prodrugs follow the same pattern: valacyclovir and valganciclovir, the l-valyl esters of acyclovir and ganciclovir, lift the oral bioavailability of their parents to roughly 60% from the 15–21% and 6–8% of the free drugs, and both are made by chemical synthesis and reconverted in vivo by a dedicated hydrolase, the biphenyl hydrolase-like protein [10].

For this class, the verdict is therefore a qualified yes: chemistry is largely required (Table 2). It is qualified because the enzymatic route is difficult but not precluded, and difficult in a way that invites the biocatalyst. The proteases and acyltransferases of Section 2 can, in principle, join an amino acid to a hydroxyl through an ester, and they offer exactly the asset chemistry lacks here: an absolute stereospecificity that would deliver the single l-ester untroubled by the racemization that attends a basic coupling. The free α-amine still resists them, a rival nucleophile in the aqueous or near-aqueous media these enzymes prefer, together with the protease’s own hydrolytic inclination toward the bond just formed. One attempt on this bond has been recorded. Subtilisin, activated for organic media, transesterified acyclovir with l-valine methyl ester in a solid-to-solid reaction, reaching yields of up to 89% at 20 mg/mL and conversions of up to 70% in preliminary experiments on a gram scale [75]. Esterification with l-valine itself gave no product—the hydrochloride salt of the methyl ester, which would have masked the nitrogen, gave none either—and with the free-base donor, the enzyme acylated its amine as well, forming polymer; the authors judged the catalyst unsuitable for scale-up and named the missing enzyme as one that accepts the free amine as an acyl donor but not as an acceptor. That experiment therefore observed the α-amine competition rather than removing it. Unlike a phosphate, then, an amino acid promoiety is a target the enzyme could plausibly reach were these two problems solved, and Section 8 returns to it among the frontiers. Section 5 takes up a further point: the free amine does double duty, driving not only solubility but recognition by PEPT1, so that the amino acid ester is at once a solubilizing and a permeability-directed prodrug, a dual character that no neutral polyol or sugar confers.

**Table 2:** Two routes to amino acid ester prodrugs: the established protect–couple–deprotect chemistry, used for the marketed l-valyl esters valacyclovir and valganciclovir, against the direct enzymatic route, attempted but not yet realized as a process.

Criterion	Chemical Route (Protect–Couple–Deprotect)	Enzymatic Route (Protease/Acyltransferase)
α-Amino group	Amine masked as an *N*-Cbz or Boc carbamate, later cleaved (hydrogenolysis or acid)	Amine left unprotected, a competing nucleophile that the enzyme acylates as well
Bond formation	Carboxyl activated (carbodiimide), then esterified onto the drug hydroxyl	Carboxyl esterified onto the drug hydroxyl directly, without activation
Reaction medium	Anhydrous organic solvent, no water, amine masked	Water kept low enough to drive synthesis: the one attempt on record ran solid-to-solid in the neat amino acid ester at 1 to 3% water, with the enzyme activated for organic solvent
Stereochemistry	Partial racemization to the unwanted d-ester, poorly carried by PEPT1	Single l-ester, free of racemization (absolute stereospecificity)
Step economy and greenness	Lower: protection, activation, and deprotection add steps and waste	Higher in principle: protecting-group-free, fewer steps, once the amine is controlled
Maturity	Established: route to the marketed l-valyl esters valacyclovir and valganciclovir	Attempted once, not realized as a process: subtilisin gave valacyclovir from l-valine methyl ester, but the donor’s free amine was acylated as well, and the authors judged the enzyme unsuitable for scale-up [75]

### 3.4. PEG and Oligo(ethylene Glycol) Carriers

**Scheme 3:**
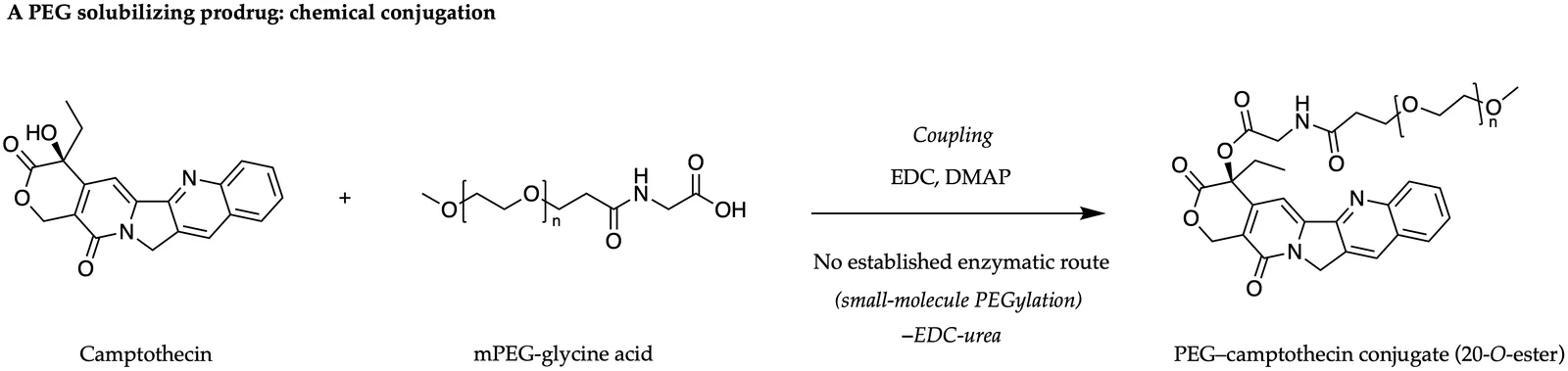
Chemical conjugation of a PEG solubilizing prodrug, shown for camptothecin. A poly(ethylene glycol) chain extended through a glycine spacer to a free carboxyl is coupled to the camptothecin C-20 hydroxyl by carbodiimide (EDC) activation with 4-dimethylaminopyridine, giving the PEG–glycinate 20-*O*-ester [77]; acylating the tertiary C-20 hydroxyl also stabilizes the α-hydroxy-δ-lactone in its active form and raises aqueous solubility by orders of magnitude [76]. A single conjugate is drawn for clarity, whereas the conjugates taken into clinical trials are multivalent: pegamotecan carries two camptothecins on a bifunctional 40 kDa PEG (a glycine spacer in [77], alanine in [76]), and EZN-2208 about four SN38 molecules on a four-arm PEG.

Poly(ethylene glycol) is the most widely used solubilizing polymer in the clinic (Scheme 3), and the aqueous-solubility gains it affords rival or exceed those of any sugar promoiety: conjugation raises the solubility of camptothecin from ~0.0025 to at least 2 mg/mL as its 40 kDa PEG ester [76], and enhances that of the practically insoluble SN38 roughly 1000-fold [77]. Yet the verdict map places PEGylation on the chemical side, for reasons that are structural rather than incidental.

In every reported solubility prodrug, the bond is forged by chemical activation. The polymer terminus is converted to an active ester (*N*-hydroxysuccinimide), an active carbonate (*p*-nitrophenyl chloroformate), or a carboxylic acid coupled through a carbodiimide (DCC/EDC), and then condensed with an –OH, –NH_2_, or –SH handle on the drug, with competing groups masked where necessary [77]. Higher-molecular-weight PEGs (of at least 30–40 kDa) are needed to slow renal clearance and, for anticancer agents, to secure the passive tumor accumulation upon which the strategy depends, while amino acid spacers such as glycine or alanine are chemically interposed to tune the hydrolysis rate of the releasable ester [76].

Biocatalysis offers no advantage here, and it offers none for the same reason that it performs well with polyols: its decisive strength is protecting-group-free regioselectivity among competing hydroxyls, and a PEG terminus is mono-functional (two in the diol form), so there are no competing sites for that selectivity to distinguish and a single chemical coupling suffices. The point of attachment reinforces this: in clinical camptothecin and SN38 conjugates, the polymer is esterified at the drug’s C-20, a sterically hindered tertiary hydroxyl whose low nucleophilicity forces even the chemical acylation to rely on carbodiimide activation or on DMAP-assisted acyl transfer [78,79,80]; such hindered tertiary alcohols are poor substrates for lipase-catalyzed acylation. The polymer adds a second obstacle, and it is not its size. Sheer size is not disqualifying: lipases esterify low-molecular-weight PEG [58] and transglutaminase or sortase attach 10 to 20 kDa PEG to proteins [81]. In those reactions, only the chain end reaches the catalytic residue while the coil stays outside in solvent. A camptothecin 20-*O*-ester instead asks one pocket to accept a 40 kDa coil and a hindered tertiary alcohol on a rigid pentacyclic core at the same time, so the steric cost compounds rather than replaces the primary objection that PEG offers no competing sites for regioselectivity to distinguish. The enzymatic advantage therefore evaporates, and in every solubility prodrug of this class reported to date, the bond to the polymer is formed chemically.

Enzymatic PEGylation is established for proteins and peptides, where transglutaminase- or sortase-mediated conjugation attaches the polymer in a site-specific manner to defined residues [81]. For the poorly soluble small molecules that are the subject of this review, the record is shorter. Three recent reports apply a lipase to a PEG-containing system, each at a different step. In the first, the enzyme builds the polymer and not the bond to the drug: CaLB polymerizes divinyl adipate with PEG400, glycerol, or diglycerol and 1,6-hexanediol to a polyester excipient, and the apparent aqueous solubility of curcumin is then raised by encapsulating it in nanoparticles of that polyester, with no bond formed between drug and carrier [82]. In the second, the enzyme does form the bond to the drug: CaLB adds doxorubicin and folic acid by Michael addition to a tetra-acrylate platform built from a PEG of 882 g/mol, although the object is folate-mediated targeting rather than solubility, and no solubility measurement is reported [83]. The division of labor is explicit in a third report: a lipase acetylates the primary hydroxyls of diglycerol selectively, the PEG is then attached by copper-catalyzed azide-alkyne cycloaddition, and nimodipine and curcumin are carried by encapsulation in the resulting amphiphile [84]. None of them establishes the step at issue here—the enzymatic formation of a cleavable bond between a poorly soluble drug and a PEG chain long enough to act as a solubilizing promoiety. At that step chemistry remains, for the present, the only demonstrated option.

The dominance of PEG is not unchallenged, and the alternative most often put forward is instructive for the argument made here. PASylation replaces the polymer with a conformationally disordered polypeptide of proline, alanine, and serine, typically 100 to 800 residues, whose expanded hydrodynamic volume retards renal filtration much as PEG does and lengthens plasma half-life by one to two orders of magnitude [85,86]. Because the chain is ribosomally encoded, it is monodisperse rather than a distribution, biodegradable rather than persistent, and has shown neither toxicity nor immunogenicity in the animal studies reported, which speaks to the anti-PEG antibody responses seen in patients [87]. The property that PASylation is reported to improve is pharmacokinetic rather than solubilizing, because in the conjugates published so far, the measured outcome is plasma half-life. Where that literature does report a solubility improvement it belongs to a protein partner, interferon-β1b or ferritin, whose aggregation the PAS chain suppresses, and no aqueous solubility value has been reported for a poorly soluble small molecule carrying a PAS chain. PASylation is therefore discussed here as the uncharged, highly hydrophilic random coil put forward to replace PEG, and not as a promoiety with a recorded solubility gain, whereas PEG holds its place in this review on the camptothecin and SN38 values given at the start of this section.

For a protein or peptide partner, the conjugate is then assembled with no chemistry at all: the polymer is fused genetically and expressed in *Escherichia coli*, so there is no coupling step and no separation of conjugated from unconjugated material. For a small molecule the position is different, though less settled than it first appears. In every reported case the polymer is attached by chemical coupling, but the reason is not that the chain resists enzymatic chemistry. The isolated PAS polymer presents a single free α-amine at its N-terminus, and engineered peptide ligases derived from subtilisin acylate precisely that group in aqueous medium, and omniligase-1 has been used on a hundred-gram scale in the synthesis of the peptide drug exenatide [88]. Sortase A, for its part, joins oligoglycine-modified non-protein molecules to polypeptides carrying an LPXTG motif, and does so for cytotoxic payloads such as monomethylauristatin E and maytansine in the manufacture of antibody–drug conjugates [89]. Applying either to a PAS chain would impose conditions rather than obstacles: peptide ligases do not accept proline at the acceptor position, so a proline-rich polymer would have to present another residue at its terminus, and the sortase route requires its recognition motif to be encoded into the chain. The missing element is therefore not a catalyst but a demonstration, and the step that would remain chemical is the activation of the drug as an acyl donor. This is a gap in what has been attempted rather than in what is possible, and it is one of the more concrete opportunities the present survey identifies (Section 8.2). Such an experiment would have to report the aqueous solubility of the isolated conjugate and not only the yield of the coupling, because whether a PAS chain solubilizes a small molecule to the degree that PEG does is not answered by the published record.

### 3.5. Ionizable Promoieties: Phosphate, Hemisuccinate, Choline

**Figure 4:**
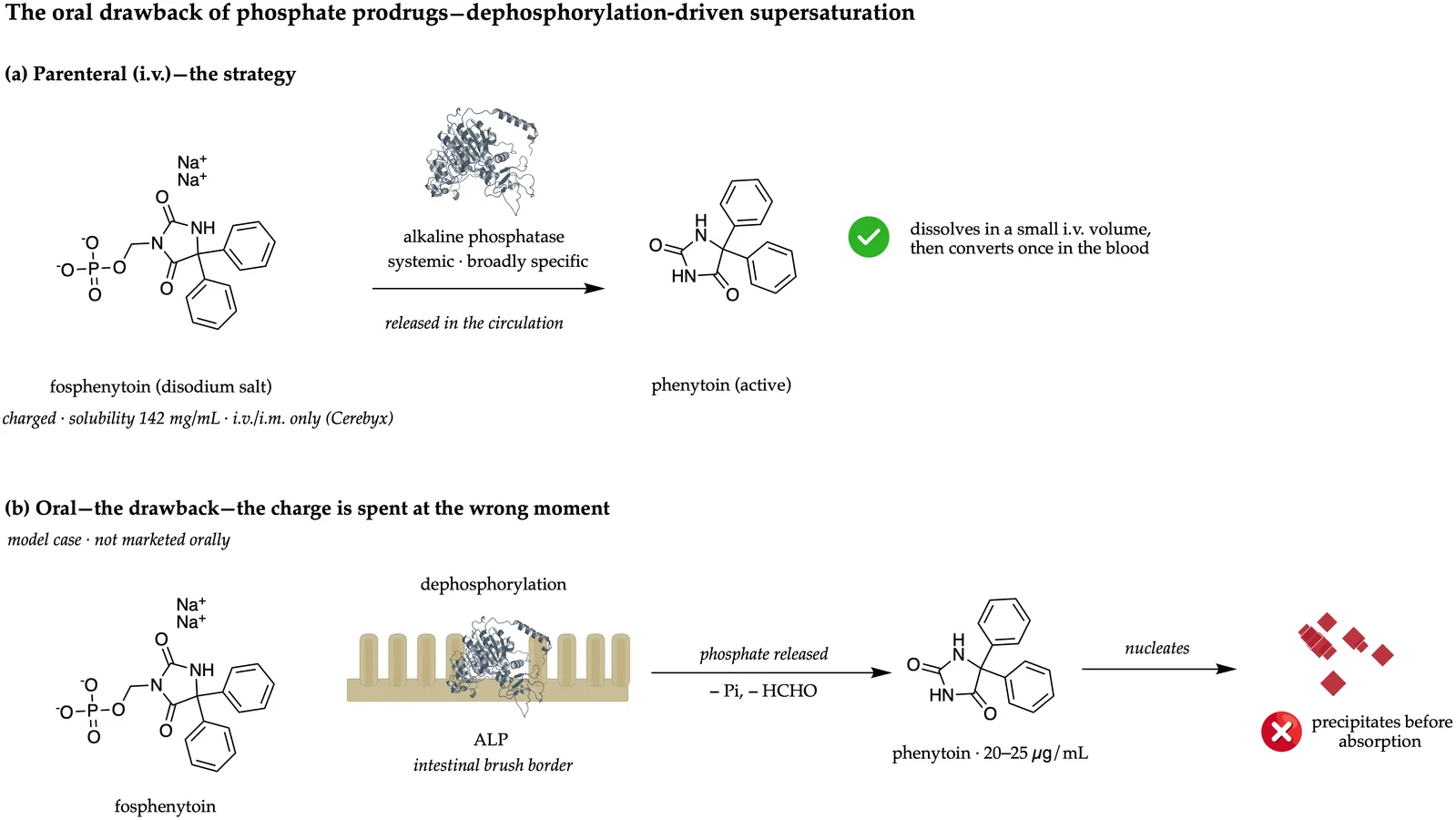
The oral drawback of phosphate prodrugs: dephosphorylation-driven supersaturation, shown for fosphenytoin. (**a**) Parenterally the strategy works: fosphenytoin, the disodium phosphate ester of 3-hydroxymethylphenytoin (142 mg/mL, intravenous or intramuscular only), is dephosphorylated in the bloodstream by alkaline phosphatase, regenerating phenytoin and releasing formaldehyde as the hydroxymethyl spacer collapses. (**b**) By the oral route, the same reaction becomes a liability: alkaline phosphatase at the intestinal brush border strips the phosphate before absorption, leaving a locally supersaturated solution of the poorly soluble parent (phenytoin, 20–25 µg/mL) that nucleates and precipitates in the lumen (Section 3.5) [90,91]. The placental isozyme (PDB 1EW2) is drawn in both bands as a representative alkaline phosphatase; the brush-border enzyme of band b is a distinct gene product.

With the ionizable promoieties, the answer to the title’s question inverts, and it inverts for structural rather than circumstantial reasons. A polyol or a sugar raises solubility by adding a neutral, hydrogen-bonding surface; a phosphate, hemisuccinate or choline ester raises it by introducing a group that carries a charge at physiological pH, and the charge delivers some of the largest solubility gains in the whole repertoire. Phenytoin, sparingly soluble at 20–25 µg/mL, becomes fosphenytoin (Figure 4), the disodium phosphate ester of its 3-hydroxymethyl derivative, since phenytoin itself carries no hydroxyl to phosphorylate, soluble at 142 mg/mL and equivalent to roughly 88 mg/mL of phenytoin: a gain of roughly 3500-fold, among the largest in the review and realized as the salt, through the ionized phosphate and its sodium counter-ions, and so not measured on the same basis as the neutral-conjugate gains of Section 3.1 and Section 3.2 [90]. It is precisely this magnitude that makes ionizable prodrugs the strategy of choice where solubility must be forced to its limit, above all in parenteral and intravenous formulations, in which the whole dose must dissolve in a small aqueous volume.

However, the bond that produces the gain is, with rare exceptions, forged by chemistry, and here the contrast with Section 3.1 is sharpest. The phosphorylation of fosphenytoin could not be achieved by direct reaction on the hydroxymethyl handle. The alcohol was poorly reactive and the phosphorylated species prone to decomposition, so the synthesis proceeded instead through a chloromethyl intermediate displaced by a protected (dibenzyl) phosphate and later freed by hydrogenolysis, or through phosphoramidite coupling, oxidation, and deprotection [90]. This is protecting-group chemistry that no biocatalyst removes the need for.

The phosphoramidite route is described here because it is the step that replaces direct phosphorylation. The reagent used by Sauer and co-workers, di-*tert*-butyl *N*,*N*-diethylphosphoramidite, is a trivalent phosphorus species carrying two *tert*-butyl-protected oxygens and one dialkylamino group. Activation by 1*H*-tetrazole converts that amino group into a leaving group, the drug hydroxyl then adds to phosphorus to give a phosphite triester, oxidation with *tert*-butyl hydroperoxide raises phosphorus to the pentavalent state, and trifluoroacetic acid removes the two *tert*-butyl groups to leave the phosphate monoester [92]. Those authors attempted both routes on two antagonists of the same series, one bearing a primary aliphatic alcohol and the other a phenol. On the aliphatic alcohol, the two routes gave similar overall yields. On the phenol, phosphorus oxychloride gave no reaction at room temperature and degraded the substrate at higher temperature, so the aryl phosphate was obtained only through the phosphoramidite route. The aliphatic prodrug of that pair, MSX-3, was carried to gram scale; it dissolves at 9 mg/mL, its aqueous solution stands at pH 7 and is stable there for several hours, and alkaline phosphatase releases the parent antagonist from it. The route does not, however, remove the objection that places this class on the chemical side. The protecting groups sit on the phosphorus reagent rather than on the drug, so no regiochemical masking of the substrate is required when it carries a single hydroxyl, as both of these did. A deprotection step is nonetheless unavoidable, and, where a drug carries more than one hydroxyl, the activated reagent is intercepted by whichever is accessible, so the masking problem returns. The reagent quantities are correspondingly large: the published preparation uses five equivalents of the phosphorylating agent, twelve of the activator, and one hundred and forty of the oxidant, in dried solvents under nitrogen, with column chromatography to isolate the protected triester.

On the enzymatic side, the position has recently changed, and it has changed for phenols rather than for drugs. PsiK, the kinase that phosphorylates psilocin in psilocybin biosynthesis, accepts a range of substituted phenols and benzenediols beyond its native substrate, and active-site variants widen that range further; one substrate was carried to 1.52 g of product in a single reaction with ATP regenerated from inexpensive inorganic polyphosphate [93]. Its substrates, however, are model phenols and benzenediols rather than drugs, no solubility of a phosphorylated product is reported in that work, and its authors close by naming the phosphorylation of drug-like small molecules as the aim of further engineering rather than as an outcome of it. Phosphorylation of a phenol is therefore now within reach of an engineered kinase, while the purpose served here by the phosphoramidite route, raising the aqueous solubility of a poorly soluble drug, has not yet been attempted with one. This is the most sharply defined of the openings identified in Section 8.2.

Beyond that one engineered exception, kinases are the wrong tool for an arbitrary drug on two counts: they consume ATP as the phosphoryl donor, and their substrate recognition is narrow, evolved for particular metabolites rather than promiscuous across chemical space. That objection holds for kinases, and the biocatalysis literature states it in the same terms: the cofactor can be recycled, but a separate enzyme is required for each class of substrate converted [94]. It does not hold for a second class of enzyme. Bacterial nonspecific acid phosphatases are hydrolases operated in the synthesis direction [95]. A conserved active-site histidine accepts the phosphoryl group from the donor to give a phosphoenzyme intermediate; a second histidine then activates a nucleophile, and whether that nucleophile is water or an alcohol decides between hydrolysis and transfer to the alcohol [96]. The donor is pyrophosphate rather than ATP, so nothing has to be regenerated, and the transfer is regioselective: PhoN from *Shigella flexneri* phosphorylates glucose to glucose-6-phosphate with no glucose-1-phosphate formed, and inosine to the 5′-monophosphate alone [94]. The scale is industrial, and in each case it is reached with cells rather than with isolated protein. *Escherichia coli* overproducing an evolved *Morganella morganii* phosphatase gives 101 g/L of inosine 5′-monophosphate at 88% molar yield [97], a *Pseudomonas aeruginosa* acid phosphatase, expressed in cells, gives 50 g/L of ascorbyl 2-phosphate in a 7.5 L reactor [98], and an engineered *Salmonella typhi* phosphatase, co-expressed with a second enzyme, gives 14.5 g/L of pyridoxal 5′-phosphate [96]. The immobilized enzyme has also been run in fed-batch and continuous packed-bed reactors, delivering six different phosphate esters on a gram scale [99].

Three measured constraints nonetheless keep this chemistry away from the substrates of the present review. The first is the acceptor concentration the enzyme requires. Michaelis constants for the acceptor run from 5.3 mM for glucose to 192 mM for inosine [94], 117 mM for inosine with the native *Morganella* enzyme and 43 mM after two rounds of directed evolution [97], 93 mM for ascorbate [98] and, for pyridoxine, of the order of 100 mM in the wild-type enzyme and in each of its engineered variants [96], and the preparative glycerol phosphorylations are conducted at 55 to 80% substrate by weight [100]. Phenytoin dissolves at 20 to 25 µg/mL, which is 0.08 to 0.10 mM. A poorly soluble drug therefore presents an acceptor concentration between sixty and two thousand times below these constants, so the reaction would run far from saturation, at a rate proportional to that concentration; working below the Michaelis constant is not by itself an obstacle, but at that concentration the product titer would be about 0.03 g/L against the 14 to 101 g/L at which these enzymes are operated, and it is the titer that decides whether a process is practicable. The same limit is reported from within the field: the solubility of inosine reached only two thirds of the Michaelis constant of the native enzyme [97]. The second constraint is the position of the equilibrium. The enzyme that forms the ester also hydrolyzes it, and it binds the phosphorylated product more tightly than the acceptor, the reported Michaelis constants being 5.3 mM against 0.02 mM for the glucose pair and 192 mM against 0.30 mM for the inosine pair [94]; the native *Morganella* enzyme was judged unsuitable for the phosphorylation of nucleosides until directed evolution lowered its nucleotidase activity to about one sixth of the wild-type V_max_ rather than abolishing it [97]. The third constraint is the acceptor itself. Every acceptor reported to date for this chemistry is a small, freely soluble alcohol, primary in every case except the enolic 2-hydroxyl of ascorbate: glucose, nucleosides, dihydroxyacetone, glycerol, *N*-acetylglucosamine, allyl alcohol, ascorbate, and pyridoxine [99]. None is a drug, and none of these reports is directed at solubility: the products are flavor enhancers for food, a stabilized derivative of vitamin C, a vitamin B6 cofactor, and phosphorylated building blocks for aldolase cascades. Where aqueous solubility appears, in two of the six reports, it does so as a limit on the reaction rather than as its object.

Phenytoin, with which this section opened, carries no hydroxyl at all, so the hydroxymethyl handle that fosphenytoin phosphorylates has to be installed chemically before any enzyme could act upon it. Chemistry remains required for the substrates with which this review is concerned, and no enzymatic route to a water-soluble phosphate prodrug has been reported. The reason is not that a promiscuous phosphotransferase is unavailable, since one exists and operates at industrial scale, but the three measurable quantities set out above: the acceptor concentration the enzyme demands, the equilibrium against which it has to be driven, and the handle it requires and a poorly soluble drug frequently lack.

One asymmetry qualifies this verdict without softening it: the phosphate bond is made by chemistry but cleaved by an enzyme, and the enzyme that cleaves it is far less selective than the chemistry that makes it. Alkaline phosphatase is broadly specific and abundant, at the intestinal brush border and throughout the body, so the prodrug that only chemistry can build is reconverted to a drug with ease, a division of labor opposite to that of the neutral esters, where a single hydrolase both forms and cleaves the bond. But this very generosity carries a liability that belongs to *Pharmaceutics* and returns in Section 5. Because dephosphorylation is fast, an orally administered phosphate prodrug can dissolve, be stripped of its charge at the brush border, and generate a locally supersaturated solution of the poorly soluble parent drug that precipitates before it is absorbed (Figure 4). Heimbach and co-workers showed this directly for fosphenytoin, TAT-59, and estramustine phosphate: enzyme-mediated dephosphorylation drives supersaturation, and the induction times for precipitation can fall within gastrointestinal residence times under realistic conditions [91]. The phosphate strategy therefore succeeds parenterally, fosphenytoin being an intravenous agent, and so often fails by the oral route: the solubility is real, but the charge that confers it is removed before the drug has been absorbed.

The exception among the three compounds Heimbach and co-workers studied is explained by the same mechanism. Estramustine phosphate is one of the few phosphate prodrugs marketed for oral use, and it escapes precipitation not because it is cleaved slowly but because of what surrounds the parent at the moment of release. Dephosphorylation is progressive, so the estramustine appearing at the brush border does so in a lumen that still holds most of a 400 to 1000 mg dose of unconverted prodrug, and that prodrug binds and solubilizes it: estramustine’s solubility rises roughly forty-fold, from 0.001 to 0.04 mg/mL at pH 7.4, in its presence. Since the driving force for nucleation is the ratio of the released concentration to that solubility, raising the denominator forty-fold keeps supersaturation low and the induction time beyond gastrointestinal residence times, whereas no comparable effect was found for fosphenytoin or TAT-59. The solubilization is a physical, non-covalent interaction between prodrug and parent in solution, distinct from the covalent conjugation that is this review’s subject [91].

The hemisuccinate and choline esters follow the same pattern. A hemisuccinate adds a terminal carboxylate by acylation with succinic anhydride, and a choline ester adds a quaternary ammonium. both are ionizable, both are made chemically, and both share the phosphate’s defining character, a large, charge-driven solubility gain reached by a route for which no general biocatalytic equivalent exists. They confirm rather than complicate the reading of this class: where the promoiety is ionizable, the bond is made by chemistry, and the verdict map turns, for the first time, decisively red.

**Scheme 4:**
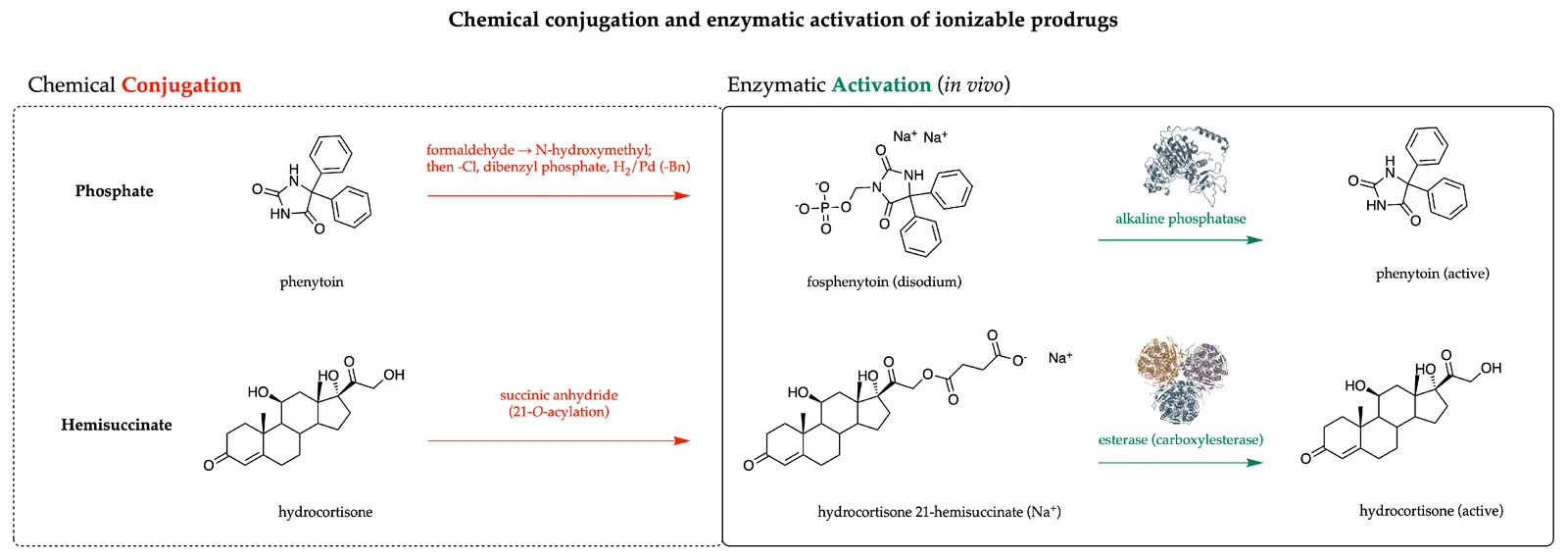
Chemical conjugation and enzymatic activation of the two covalent ionizable classes of Section 3.5. Top, phosphate: Phenytoin is *N*-hydroxymethylated (formaldehyde) and phosphorylated by the chloromethyl/dibenzyl-phosphate route, freed by H_2_/Pd hydrogenolysis, to fosphenytoin, its disodium phosphate ester; in vivo, alkaline phosphatase (human placental ALP, PDB 1EW2) cleaves the P–O ester, regenerating phenytoin with release of phosphate and formaldehyde [90]. Bottom, hemisuccinate: The primary 21-hydroxyl of hydrocortisone is acylated by succinic anhydride to the 21-hemisuccinate (sodium salt), which a carboxylesterase (human CES1, PDB 1MX1) hydrolyzes in vivo, releasing hydrocortisone and succinate. The 21-hemisuccinate design and its solution-stability limits are documented for corticosteroid 21-esters generally, the experimental series being methylprednisolone [101]; the activating esterase is assigned by class, not by that study.

Concrete cases bear this out (Scheme 4). The hemisuccinate is the classic solution-stable, water-soluble design for poorly soluble drugs: the 21-hemisuccinates of corticosteroids, marketed as their sodium succinate salts (hydrocortisone and methylprednisolone sodium succinate), dissolve readily for intravenous use and are cleaved within minutes by esterases, at the recognized cost of limited solution stability [101]. A choline-based promoiety raises solubility further: cholinium salts of methotrexate are more than three orders of magnitude more soluble than the free drug, though here the gain comes from ion pairing rather than covalent conjugation, so this is strictly an ion-paired salt and not a covalent prodrug [102].

### 3.6. Beyond Esters: The Linkage Palette and Its Cleavage Chemistry

The esters that dominate the preceding sections are only the most common members of a broader palette of solubilizing linkages, and the choice of bond is not incidental: it sets both the chemical stability of the prodrug on the shelf and the mechanism and rate of its reversion in the body [103]. Esters, carbonates, carbamates, glycosidic bonds, and phosphate esters differ systematically along these two axes. Esters are the most versatile and the most readily cleaved, hydrolyzed chemically and enzymatically by the ubiquitous carboxylesterases (Section 5.3) [104,105]. Carbonates and carbamates are cleaved less readily, the carbamate slowly enough that in bambuterol, two phenols of terbutaline are masked as dimethylcarbamates to avoid rapid first-pass metabolism, which is useful for when premature cleavage must be avoided but which demands a more active enzyme, or a self-immolative spacer, to release the drug [10,106]. Glycosidic bonds are chemically robust and are cleaved selectively by glycosidases, a property exploited for site-directed release (Section 3.2). Phosphate esters are chemically stable, afford some of the largest solubility gains because the group they carry is charged at physiological pH, and are rapidly cleaved by the broadly distributed alkaline phosphatases, which can precipitate the parent drug when activation is too rapid (Section 3.5 and Section 5.2) [91].

**Scheme 5:**
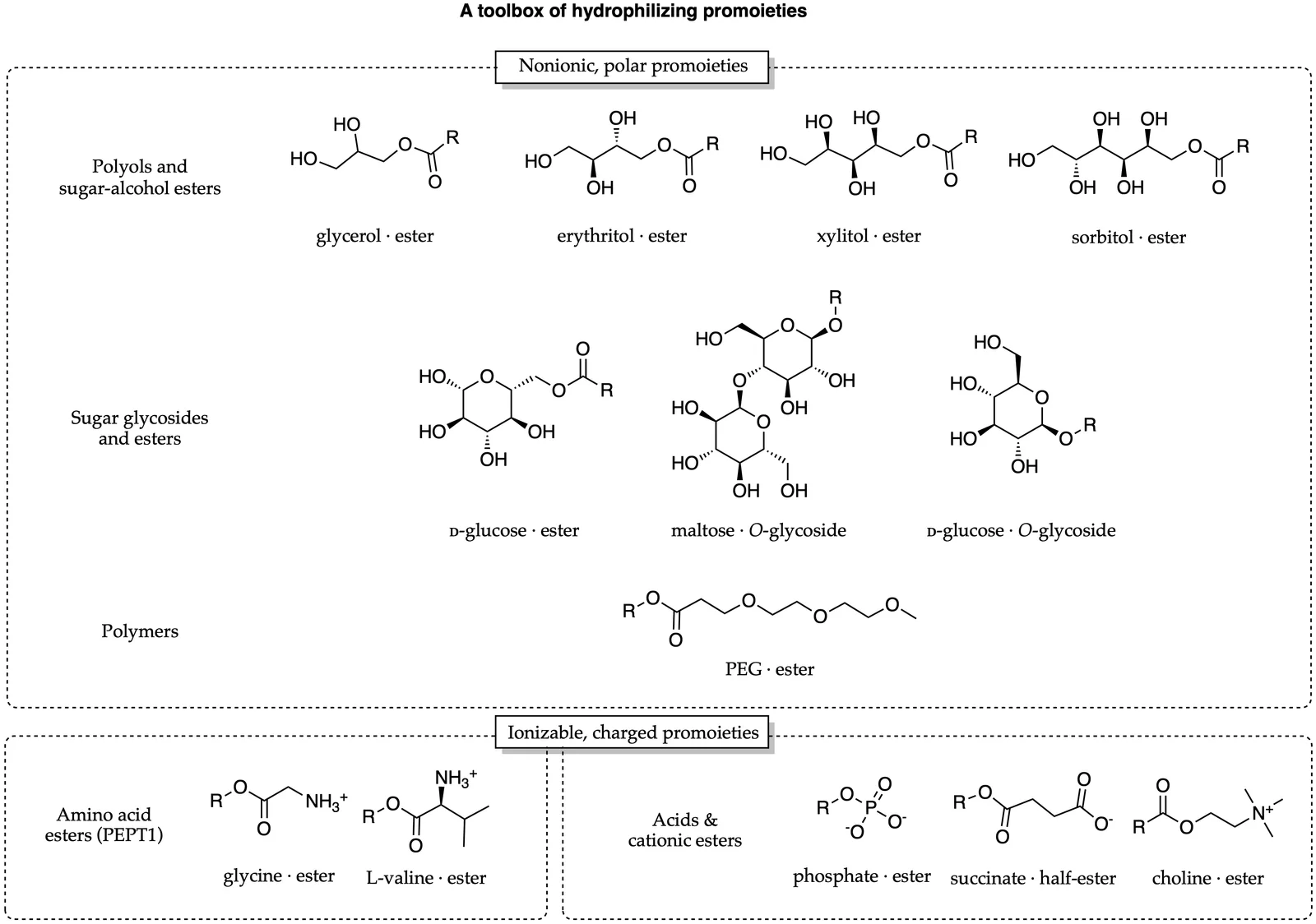
A toolbox of hydrophilizing promoieties, drawn independently of the drug they modify: each is the fragment appended to the parent drug, with R the drug and the linkage and any charge shown explicitly. Nonionic, polar promoieties (top: polyol and sugar-alcohol esters, sugar glycosides and esters, and PEG) raise aqueous solubility by adding a hydrogen-bonding surface and, for the polyols and sugars, are accessible to enzymatic conjugation. Ionizable, charged promoieties (bottom) raise it by introducing a charge and are installed chemically, their largest gains realized as salts: amino acid esters (a cationic α-amine; PEPT1 substrates), phosphate and hemisuccinate esters (anionic), and choline esters (a permanent cation). The polyol esters are drawn in one of their possible configurations.

The practical lesson is that the linkage is a design variable to be matched to the promoiety and to the intended site of action, not a default. For the enzymatic hydrophilization that is this review’s subject, the ester and the glycosidic bond are the natural targets: both are formed well by the hydrolases and transferases of Section 2 and cleaved cleanly by widely available enzymes, whereas the carbamate and the phosphate, attractive though they are for stability or for the magnitude of the solubility gain, still belong largely to chemistry both to build (Section 3.3, Section 3.4 and Section 3.5) and, for the carbamate, to release. Reading the palette this way closes the loop between how a bond is made (Section 2, Section 3 and Section 4) and how it is broken (Section 5). Representative structures for each promoiety class are collected in Scheme 5.

## 4. Molecular Determinants of Selectivity and Rational Design

### 4.1. Regio- and Stereoselectivity: Discriminating Hydroxyls and Controlling the Anomeric Center

If Section 3 established that enzymes can build hydrophilizing bonds, this section explains why they build them so well, and the answer is selectivity, the property that the three-part test of Section 1.3 placed at the center of the comparison. The defining difficulty of grafting a polar promoiety is control over both the degree and the site of substitution: a polyol or a sugar presents several hydroxyls of near-identical reactivity, and the enzyme’s value lies in acylating one of them, once, without protecting the rest. That lipases can do this was established early and generally. Several unrelated lipases catalyze the transesterification of monosaccharides with activated esters in pyridine or dry organic media, acylating the primary 6-hydroxyl of glucose, mannose, and other sugars selectively enough to give single 6-*O*-acyl products [107]; further, once that primary position is blocked, lipases discriminate among the four remaining secondary hydroxyls of C-6-protected glucose, galactose, and mannose, with different lipases favoring different positions so that even C-2 or C-3 monoesters become accessible through the choice of enzyme alone [108]. This is exactly the protection-free regiocontrol that the chemical route can reach only through protection and deprotection (Section 6.3), and it carries over directly to the polyol prodrugs of Section 3.1, where the same primary-hydroxyl selectivity lets CaLB attach ibuprofen, or ferulic acid, to a single position of xylitol [53,60].

Control of the second axis, the degree of substitution, is documented in the same series by the absence of the diester: under optimized conditions, the ibuprofen–xylitol, sorbic acid–glycerol, and (*S*)-ibuprofen–glycerol esterifications gave no detectable diester, and the xylitol monoferulate synthesis only traces of the bis(ferulate) [53,60,62,65]. Raising the polyol, at acid-to-polyol ratios of 1:3.5 and 1:6, lowered the conversion; the condition adopted is a three-fold excess of ibuprofen over erythritol, with diacylation held down by running at 50 °C, the lowest temperature tested. Where that suppression is not applied, the diacylated ibuprofen–erythritol ester becomes a second product, and the study separates the two by column chromatography and reports product yields of 75% for the monoester and 50% for the diester [62]. That the diester is the outcome to be avoided rather than a harmless companion follows from its properties, since it is more lipophilic than the monoester and, for erythritol, a solid where the monoesters are oils [60,62]. The same secondary-hydroxyl discrimination operates on drug scaffolds: CaLB selectively acylates the 3α- over the 7β-hydroxyl of the bile acid ursodeoxycholic acid, and can subsequently remove the 3α ester selectively, distinguishing two secondary hydroxyls on one steroid nucleus [109].

Regiocontrol is only one axis; stereocontrol is a second, independent one, and it matters most for the glycosides of Section 3.2. Glycosyltransferases set the anomeric configuration of the new bond, and that configuration follows from the mechanism rather than from the engineering: a glucansucrase is a retaining enzyme working from sucrose, so the sugar arrives as an α-glucoside, whereas the naturally occurring glycosides are typically β. What engineering widened in the glucansucrase GTF-D from *Streptococcus mutans*, variant M4, is the range of acceptors, which now includes flavonoids such as catechin, genistein, and daidzein glucosylated from cheap sucrose, so the enzyme delivers products new to the natural-product reservoir, with solubility and bioactivity that must be characterized afresh, the major monoglucosides being accompanied by minor diglucosides [49]. A third axis, enantioselectivity, operates on the drug rather than the promoiety: CaLB esterifies the (*R*)-enantiomer of ibuprofen preferentially, as docking predicted and as the characterization of the products isolated from the racemate confirmed, so the biocatalyst also filters the stereochemistry of the parent acid it conjugates [62]. Regio-, stereo-, and enantioselectivity are therefore three distinct handles set in a single step by the choice of enzyme, and, as Section 5.3 notes, the anomer fixed here also decides which glycosidase can later cleave the bond in vivo.

### 4.2. Rational Design: Molecular Docking, Near-Attack Conformations, Enzyme Engineering

Selectivity of this precision is not accidental, and it can increasingly be predicted before a single reaction is run. Its conceptual basis is the near-attack conformation (NAC): the fraction of enzyme–substrate complexes populated in a geometry already poised to reach the transition state governs the observed rate, so a hydroxyl, or an enantiomer, that more readily adopts a productive near-attack geometry reacts faster and more selectively [110]. Molecular docking operationalizes this idea by ranking, for a given substrate pose in the active site, which hydroxyl or which enantiomer sits in a near-attack geometry. Applied to the ibuprofen–polyol series, docking of the two ibuprofen enantiomers into the lipase active sites predicted opposite enantiopreferences for the two lipases—CaLB favoring the (*R*)-acid, a prediction that the products isolated from the racemate then confirmed—and rationalized the regiochemistry through the near-attack geometries accessible to each pose (Section 3.1) [62]. Docking therefore allows the selectivity to be predicted before the reaction is run rather than recorded after it. The same computational–experimental workflow extends beyond ibuprofen: docking of nine cinnamic-acid derivatives predicted how ring substituents and the degree of unsaturation reshape the productive binding poses on CaLB, in agreement with the measured conversions [64].

Where the natural active site does not offer the required geometry, it can be reshaped, and the same engineering logic met in Section 2.3 becomes a rational-design tool here: site-saturation mutagenesis of GTF-D produced a variant with markedly improved transglucosylation of flavonoids, an active site redesigned to admit new acceptors and to set the anomeric outcome [49]. Read together, docking and NAC analysis (which explain and predict selectivity) and directed or semi-rational engineering (which introduce it where it is absent) form the design loop that will decide whether the enzymatic route can be extended to the substrate classes where chemistry still dominates (Section 3.3, Section 3.4 and Section 3.5 and Section 8.2).

### 4.3. Structural Verification: NMR-Led Assignment of Acylation Position and Degree of Substitution

A claim of regioselectivity is only as good as the evidence that locates the new bond, and here nuclear magnetic resonance is decisive. The position of acylation is established most directly by heteronuclear multiple-bond correlation (HMBC), which reveals the long-range ^1^H–^13^C coupling between the proton on the esterified carbon and the ester carbonyl, pinpointing which hydroxyl of the polyol or sugar carries the acyl group; a full one- and two-dimensional suite (^1^H, ^13^C, DEPT, COSY, HSQC/HMQC, and HMBC) with mass spectrometry then confirms structure and, for polyols, the degree of substitution. This is the approach by which the ester position was assigned historically for enzymatic sugar monoesters via ^13^C NMR [107], and by which the site of esterification was fixed and a single regioisomer confirmed for both the ibuprofen–xylitol prodrug and xylitol monoferulate [53,60]. Without HMBC, or an equivalent long-range correlation, the regiochemistry is asserted rather than demonstrated. Across the seventeen primary studies audited in Section 6.4, the site of attachment is established by NMR evidence in twelve of them, and the exceptions are informative rather than general: for a symmetrical polyol such as glycerol, erythritol, or xylitol, a demonstration of primary-hydroxyl selectivity settles the constitution completely because the two primary positions are constitutionally equivalent and differ only in configuration, a difference a chiral catalyst may still make, whereas for an unsymmetrical polyol such as sorbitol it does not, C-1 and C-6 being distinct positions that give distinct products.

The spectroscopy that verifies a product can, increasingly, also follow the reaction that makes it. In-line and on-line NMR (now feasible on benchtop instruments and in flow, and quantitative down to the microliter scale) turns NMR from an endpoint characterization tool into a real-time monitor of conversion, kinetics, and reaction optimization [111]. For a hydrophilizing esterification, this means that the synthesis-versus-hydrolysis balance (Section 2.4) and the emergence of the target regioisomer could be tracked as they occur; it is the natural bridge from the molecular characterization of this section to the process-analytical control of Section 6.6, where the analytical method that proves the bond in the laboratory becomes the one that governs it on the plant.

## 5. From Molecule to Medicine: Pharmaceutical and Pharmacokinetic Consequences

### 5.1. Quantifying the Payoff: Solubility and Dissolution Gains

The most immediate measure of a hydrophilizing prodrug is the size of the solubility gain it delivers, and across the enzymatic conjugates of Section 3, these gains are large but far from uniform, spanning roughly 4-fold to 5500-fold for the drug conjugates—that is, from well under one to nearly four orders of magnitude—and larger still for the phytosterol esters (Table 3). Their magnitude depends less on the enzyme than on how insoluble the parent is and on how thoroughly the appended sugar disrupts its crystal lattice.

Three cautions keep these numbers meaningful. First, they are not measured on a common footing: the determinations are made in water or in buffer, at temperatures that are not always stated, by methods ranging from filtration with chromatographic quantification to turbidimetry, and the phytosterol entry compares a conjugate measured here with a parent value taken from earlier literature rather than alongside it, so fold-changes drawn from different studies should be read as orders of magnitude rather than ranked against one another. Second, where a value is reported it refers to equilibrium (thermodynamic) aqueous solubility, not to the dissolution kinetics that actually govern oral absorption; dissolution-rate profiles are rarely reported for these conjugates, so a large equilibrium gain should not be read automatically as a proportional gain in dissolution or exposure. Third, a solubility gain is useful only if the parent is released and remains active, a point developed in Section 5.2 and Section 5.3; it is why the docetaxel galactoside, released to only 45% where its glucoside reached 88%, was the least cytotoxic of the three conjugates [39].

A fourth observation concerns the endpoint on which this review turns. Of the seventeen primary studies of Table 3, six report no solubility measurement for any conjugate they made, one of them stating only that the product is more hydrophilic than the parent and one anticipating rather than measuring the gain. A further conjugate is likewise unmeasured, although the study reporting it measured a companion conjugate from the same series and is therefore scored as measured. Eleven of seventeen (65%) therefore quantify the property the conjugate was made to improve, the study-by-study scoring being given in Supplementary Table S1. The same audit shows one study in seventeen reporting an in vitro permeability surrogate and none reporting in vivo data, the asymmetry set out in Table 4 and counted in Table 6. Synthetic novelty has been easier to publish than the measurement that would justify it, and the pattern runs parallel to the green-metrics gap of Section 6.4.

**Table 3:** Representative enzymatically and chemo-enzymatically synthesized hydrophilizing prodrugs, with polyol esters (Section 3.1) followed by the sugar and glycoside conjugates (Section 3.2). Columns give the promoiety and linkage, the biocatalyst and its source or form, the glycosyl or acyl donor and reaction medium with the conversion or yield where stated, the aqueous-solubility gain reported in the cited primary study (fold-increase or absolute value; n.r. = not reported, which applies to seven entries drawn from six of the seventeen studies), and notes on selectivity, activation, or retained activity. All gains are for the conjugate itself and are not comparable with the salt-form values discussed in Section 3.3, Section 3.4 and Section 3.5.

Drug/Bioactive	Promoiety and Linkage	Biocatalyst (Source/Form)	Donor/Reaction Medium	Solubility Gain	Selectivity, Activation, and Notes	Ref.
Ibuprofen	Xylitol ester (primary –OH)	*CaLB* (Novozym 435, immob.); ×5 reuse	2-methyl-2-butanol; 80% conv.	≈12× (method not stated)	Regioselective for the primary –OH	[60]
Ibuprofen	Sorbitol ester	Porcine pancreatic lipase (free)	Biphasic hexane/water; 64–73% conv.	n.r.	–	[16]
(*S*)-Ibuprofen	Glycerol ester	*Rhizomucor miehei* lipase (immob.)	Solvent-free (glycerol as substrate + stabilizer); 83 ± 5% conv.	n.r.	–	[62]
Ferulic acid	Xylitol ester	*CaLB*; >98% conv.	*tert*-amyl alcohol	n.r.	Bifunctional antioxidant conjugate	[53]
Ibuprofen	Erythritol ester	*CaLB* (Novozym 435, immob.)	2-methyl-2-butanol; 82 ± 4% conv.	463 µg/mL (≈22×; turbidimetric)	Pair of diastereomers; bio-based polyol	[62]
Cinnamic acids (nine derivatives)	Erythritol ester	*CaLB* (Novozym 435, immob.)	Solvent-assisted or solventless; yields >95%	n.r.	Docking rationalizes substituent effects	[64]
Ursodeoxycholic acid	Glyceric ester (monoglyceride)	*CaLB* (Novozym 435, immob.)	Solvent-free, glycerol as reagent and medium	123 mg/L (≈6×; UDCA 20 mg/L)	C-24 ester; primary glycerol –OH assigned by NMR	[63]
Sorbic acid	Glycerol ester	*CaLB* (immob.)	Solvent-free; 101 mg isolated, 60.8% on sorbic acid	n.r.	Antimicrobial activity improved vs. *S. cerevisiae*	[65]
Bixin (food colorant)	Sorbitol ester of norbixin (methyl-ester transesterification)	*CaLB* (Novozym 435, immob.)	2-methyl-2-butanol + 20% THF, a_w ≈ 0; 50% yield	n.r.	Hydrophilicity anticipated by the authors, not measured	[67]
Phytosterols (β-sitosterol)	Sorbitol/mannitol/xylitol ester	*C. rugosa* lipase + *B. subtilis* protease	Divinyl adipate linker; *n*-hexane, then pyridine; >94% conv.	4.6–7.9 mM (parent 2.8 × 10^−6^ mM, lit.)	Two-enzyme sequence	[66]
Docetaxel	β-*d*-glucoside (carboxyethyl spacer, ester-linked)	β-galactosidase (*K. lactis* or *A. oryzae*) or β-xylosidase, both by transglycosylation, + chemical coupling	Aqueous buffer, then chemical	≈52× (27 µM)	Sugar–drug bond formed chemically; the galactoside is released least	[39]
(*S*)-Ketoprofen	Mono-/disaccharide esters (primary –OH)	*M. miehei* lipase (resolution) + *B. licheniformis* protease	Vinyl ester; pyridine/*tert*-butanol; ≤87%	n.r. (all > parent)	Released at physiological pH	[14]
Flavonoids (catechin, genistein, daidzein, silybin)	α-glucoside (*O*-glycoside)	Engineered glucansucrase GTF-D M4 (*S. mutans*)	Sucrose	Genistein ≈4×	α-anomer; no nucleotide cofactor	[49]
Mangiferin	Maltosyl-α-(1→6) *O*-glycoside	Maltogenic amylase (*P. galactosidasius*)	Maltodextrin	≈5500×	Antioxidant retained; no cofactor	[41]
Ganoderic acid A	15,26-*O*-β-diglucoside	Two *Bacillus* glycosyltransferases (Leloir), sequential	UDP-glucose	≈1024×	Two-site sequential glycosylation	[68]
Ganoderic acids F, G	Glucoside (*O*-glycoside)	*Bacillus* GT BsGT110 (Leloir)	UDP-glucose	≈89× (F); ≈97× (G)	Single-site glucosylation	[69,70]
Niclosamide	*O*-glucoside	Engineered plant glycosyltransferases (Leloir)	UDP-glucose	≈100× (physiol. pH)	Antimicrobial and antiviral activity abrogated; the authors propose use as a prodrug	[42]

### 5.2. Solubility–Permeability Interplay and Hydrophilic–Lipophilic Balance

A solubility gain is paid for in permeability. Because intestinal permeability scales with the drug’s membrane/aqueous partitioning, raising apparent aqueous solubility tends to lower apparent permeability: solubilization by cyclodextrins or surfactants reduces the free, membrane-available fraction of drug, whereas cosolvents lower its membrane/aqueous partitioning at essentially constant free fraction; by either route, the solubility–permeability interplay cannot be ignored when a solubilizing strategy is judged [112]. For a hydrophilizing prodrug, the same tension operates through the promoiety’s polarity (beyond a point, added hydrophilicity that aids dissolution begins to hinder permeation), so the target is a balance along the hydrophilic–lipophilic axis rather than maximal solubility.

The niclosamide case is the sharpest illustration: its *O*-glucoside is 100 times more soluble at physiological conditions yet shows no effect on the growth of *Staphylococcus aureus* or on the infectivity of SARS-CoV-2 and hepatitis C virus at concentrations at which niclosamide itself is active. The authors attribute the loss to the sugar preventing membrane penetration or sterically hindering the binding interactions, and conclude that such a glycoside would be useful only if it behaved as a true prodrug, shedding its sugar at the target site [42]. The framework that formalizes this limit is the BCS (Section 1.1): solubilization rescues a Class II compound but leaves a Class IV compound constrained by poor permeability [5]. A kinetic pitfall attends the largest jumps: a supersaturated solution generated on dissolution must be stabilized against precipitation, the ‘spring and parachute’ problem, or the dissolved drug precipitates before it is absorbed [113], precisely what occurs when a phosphate prodrug is dephosphorylated too rapidly at the intestinal wall [91].

### 5.3. In Vivo Activation: Enzymatic Reconversion, Release Kinetics, Interspecies Differences

A prodrug must revert to its active parent, and whether that reversion needs a catalyst different from the one that built the bond depends on the linkage. For the neutral esters that are the main subject of this review, it does not: the same hydrolase family forms the bond in vitro, driven toward synthesis by the low-water conditions of Section 2.4, and hydrolyzes it in vivo, so what changes is the thermodynamic direction, not the enzyme (a point already made in Section 3.5). Glycosides can be built by the very glycosidases that later cleave them (transglycosylation) or by distinct glycosyltransferases, whereas phosphates are the clear opposite case, forged by chemistry and cleaved by a genuinely different catalyst, a phosphatase. In vivo, esters are cleaved by ubiquitous esterases and carboxylesterases, glycosides by glycosidases, and phosphates by phosphatases, each with its own tissue distribution and kinetics. The geraniol–ferulic acid ester (Fer-Ger) shows how tissue- and species-dependent this reconversion is: it is hydrolyzed in human whole blood with a half-life of ~194 min, roughly ten times faster in rat whole blood (~20 min) and faster still in rat liver homogenate (~4 min), yet it is not hydrolyzed in rat brain homogenate nor by neuron-differentiated N2a cells, in which no free geraniol is detectable inside or outside the cells, and the intact conjugate still prevents the loss of viability caused by hydrogen peroxide, although the authors find it pro-oxidant in those same cells and caution that the protection may reflect mitochondrial hyperactivation [114].

Such interspecies differences are the rule, not the exception, and they complicate the translation of animal data to humans. For an ester prodrug of curcumin, plasma hydrolysis proceeded in the order rat ≫ human > dog, with carboxylesterase dominating in rat but multiple esterases contributing in dog and human models [115]. The human carboxylesterases are themselves distributed unevenly (CES1 predominating in liver, CES2 in intestine and several tumors), and between them they activate a long list of ester prodrugs (oseltamivir, irinotecan, capecitabine), so the choice of linkage effectively selects both the activating enzyme and the site of activation [104,105]. The same logic explains why the docetaxel glucoside is unmasked by cancer-cell enzymes more completely than its galactoside [39]: the sugar, not only the drug, dictates where and whether release occurs.

### 5.4. Reported Bioavailability and (Pre)clinical Outcomes

When the question shifts from solubility in a cuvette to performance in vivo, the evidence base becomes markedly asymmetric, and the asymmetry is itself a finding. The solubilizing prodrugs with real pharmacokinetic and clinical data are, almost without exception, of chemical synthesis. Fosphenytoin is water-soluble and cleaved by endogenous phosphatases to release phenytoin on parenteral administration [90]. Valacyclovir, the l-valyl ester of acyclovir, raises the oral bioavailability of acyclovir from ~15–21% primarily through active uptake by the intestinal peptide transporter PEPT1 [10], with improved aqueous solubility a secondary contributor, and is made by protecting-group chemistry [72]. Even the glycoside example most cited clinically, dapagliflozin, is instructive precisely because it is not a releasing prodrug: the metabolically labile *O*-glucoside phlorizin was deliberately replaced by a hydrolysis-resistant C-glucoside to prevent β-glucosidase cleavage, a chemical solution to a stability problem [116].

Against this stands the near-absence of in vivo pharmacokinetics for the enzymatically synthesized hydrophilizing prodrugs that are this review’s subject: their solubility gains are documented, but AUC, C_max_, T_max_, oral bioavailability, and half-life are seldom measured, and the reconversion data that do exist are largely in vitro (plasma or tissue homogenates, as for Fer-Ger) rather than in vivo [114]. This is not a bibliographic gap to be closed by more searching but a real gap in the primary literature: for the enzymatic route to compete on the terms that matter to a pharmaceutics readership, the field must generate genuine pharmacokinetic data, not only solubility fold-changes. The contrast is laid out in Table 4.

**Table 4:** (Pre)clinical and pharmacokinetic outcomes of solubilizing prodrugs and of the enzymatic conjugates surveyed here: the evidence asymmetry between chemically marketed prodrugs (rows 1–4, real in vivo PK) and enzymatically or chemo-enzymatically synthesized candidates (rows 5–10, in vitro only). Row 9 is included for its reconversion data: the geraniol–ferulic acid ester was made to load lipid microparticles rather than to raise aqueous solubility.

Drug	Prodrug	Promoiety/Linkage	Route (chem/enz)	Key PK/Bioavailability	Status	Ref.
Phenytoin	Fosphenytoin	Phosphate ester (ionizable)	Chemical	Cleaved by endogenous phosphatases; parenteral/IV	Marketed	[90]
Acyclovir	Valacyclovir	*l*-valyl ester (amino acid)	Chemical	Oral F ↑ from 15 to 21% to ~60% (PEPT1-mediated)	Marketed	[10,72]
Ganciclovir	Valganciclovir	*l*-valyl ester (amino acid)	Chemical	Oral F ↑ from 6 to 8% to ~60%	Marketed	[10]
Phlorizin (parent scaffold)	Dapagliflozin	*C*-glucoside (hydrolysis-resistant)	Chemical	Metabolically stable *C*-glucoside; oral SGLT2 inhibitor	Marketed	[116]
Docetaxel	7-propionyl-docetaxel 3″-*O*-β-*d*-glucoside	Ester-linked glucoside	Chemo-enzymatic	In vitro only; hydrolyzed by cancer-cell enzymes; no in vivo PK	In vitro	[39]
Niclosamide	Niclosamide *O*-glucoside	Glucoside	Enzymatic	In vitro only; parent activity abrogated; no in vivo PK	In vitro	[42]
Mangiferin	Maltosyl-α-(1→6)-mangiferin	Maltoside	Enzymatic	In vitro only; antioxidant retained; PK n.d.	In vitro	[41]
Ganoderic acid A	GAA 15,26-*O*-β-diglucoside	Diglucoside	Enzymatic	In vitro only; PK n.d.	In vitro	[68]
Ferulic acid (Fer-Ger)	Geraniol–ferulic acid ester	Ester (bifunctional)	Enzymatic	In vitro hydrolysis only: human-blood t½ 194 min; no in vivo PK	In vitro	[114]
Ibuprofen	Ibuprofen–xylitol	Polyol ester	Enzymatic	No in vivo PK reported	In vitro	[60]

We cannot say which of two explanations that absence supports. The first is a limitation intrinsic to neutral conjugates. The second is that no one has invested in developing them. Two facts favor the second. When a drug is ionizable, industry usually solves solubility with a salt, which costs less and alters the properties of the drug without modifying its chemical structure [117]; of the 1356 chemically well-defined active ingredients listed in the FDA Orange Book, 48.6% are used in a nonsalt form and the remaining 51.4% are salts, 38.6% of basic and 12.8% of acidic molecules [118]. The intravenous ibuprofen product approved for patent ductus arteriosus is the l-lysine salt, and its pharmacokinetics were measured in 54 preterm infants [119]. The second fact is regulatory. Under 21 CFR 314.3, an ester does not change the active moiety of a drug, so an ester conjugate of an approved drug earns none of the five-year exclusivity that 21 CFR 314.108 gives a new chemical entity, whereas a non-ester covalent conjugate does [120]. Lipases make esters, and twelve of the seventeen conjugates in Table 3 are esters against five *O*-glycosides. The first explanation is supported as far as it goes, because a neutral carrier cannot match an ionizable one (Section 1.2), but it limits how large the gain can be, not whether it can be measured. One conjugate taken through an animal study would settle the question.

### 5.5. Promoiety Safety, Released Metabolites, and Activation Selectivity

The safety of a prodrug includes the fate of the promoiety it releases, and here the neutral carriers of Section 3 hold an advantage. Polyols and simple sugars are, as a class, generally recognized as safe: erythritol, xylitol, sorbitol, and mannitol carry established acceptable daily intakes, their main documented liability being dose-dependent osmotic and laxative gastrointestinal effects at high intake rather than systemic toxicity [121,122]. A polyol or sugar promoiety therefore returns, on cleavage, a metabolite with a benign and well-characterized profile, an advantage the more novel glycosides do not automatically share, since an α-glucoside or diglucoside new to nature has, by definition, no established safety record.

Activation selectivity is the second safety axis. Because the activating enzymes are distributed unevenly, CES2 and certain glycosidases being over-represented in tumor tissue, a linkage can, in principle, be chosen so that the drug is released preferentially at its target rather than prematurely in the systemic circulation, a selectivity already exploited by carboxylesterase-activated anticancer prodrugs [104,105]. Conversely, premature systemic hydrolysis wastes drug and can expose off-target tissues. Underlying all of this is a regulatory reality that enthusiasm for ‘green’ synthesis should not obscure: a prodrug is a new substance with its own toxicology, pharmacokinetics, and development burden, however efficiently or sustainably its bond was formed [31,123].

## 6. Process Translation and Green Chemistry

### 6.1. From Batch to Continuous Flow: Packed-Bed Reactors, Productivity, and Stability

With the biocatalyst chosen and immobilized, the question of Section 6 is whether the chemistry of the preceding sections can be turned into a process, and the first lever is reactor format. Moving from stirred batch to continuous flow, typically a packed-bed reactor filled with immobilized enzyme, lets the substrate stream percolate through a fixed catalyst bed: the enzyme is retained and reused, product is separated from catalyst simply by leaving the column, and residence time and mass transfer can be tuned precisely [124,125]. The case that established the format here is a lipophilizing one: a continuous-flow packed bed of immobilized CaLB (Novozym 435) converts geraniol and propionic acid to geranyl propionate at about 87% conversion in a 15 min residence time, with Novozym 435 the most active and stable of the biocatalysts screened [126]. Geranyl propionate is a fragrance ester, and it is cited here for the reactor format alone, because no continuous-flow synthesis of a water-soluble prodrug has been reported. The flow work on drug esters that does exist was done to resolve racemates. Immobilized *Candida rugosa* lipase forms the 2-(*N*-morpholino)ethyl ester of ibuprofen in cyclohexane in a packed bed run for 208 h at space times of 0.28 to 1.50 h, but the object of that study was the (*S*)-enantiomer, the rationale it gives for making the ester is masking the acid and raising skin permeability, and it reports no aqueous solubility [127]. The syntheses of Section 3 are themselves all stirred batch, so transfer of the packed-bed format to the polyol, sugar, and glycoside classes is an expectation rather than a result. The same arrangement, immobilized CaLB in a packed bed, would be the natural format for those classes, and the operational stability and reusability that make it economic are properties of the immobilized preparation discussed in Section 2.5 [58].

### 6.2. Scale-Up, Biocatalyst Economics, and Productivity Metrics

Whether such a process is viable is ultimately an economic question, and the decisive quantity is the biocatalyst yield, the mass of product obtained per mass of enzyme consumed. Techno-economic analysis puts this in perspective: for large-volume, low-value products, a biocatalyst must typically deliver of the order of 2000–10,000 kg of product per kg of immobilized enzyme to be economic, whereas for high-value pharmaceuticals, a yield of only 50–100 kg of product per kg enzyme can already suffice [128]. Hydrophilizing prodrugs are high-value, low-volume products and therefore tolerate a higher biocatalyst cost per kilogram, so an immobilized, reusable lipase, even a comparatively costly one, can be amortized over many cycles [58]. High enzyme loading, the recognized brake on biocatalytic economics, consequently matters less here than it would for a commodity chemical [17,21].

Reporting such economics consistently, however, requires the metrics that industry actually uses, biocatalyst yield (kg product per kg enzyme), turnover number, space-time yield (volumetric productivity), and the enzyme’s share of the cost of goods, rather than conversion alone [128]. These are the quantitative counterparts of the qualitative green claims examined next, and the enzymatic-prodrug literature rarely reports them, the measurement gap that Section 6.4 identifies.

### 6.3. The Green Case, Qualitatively, and the Chemical Benchmark It Is Measured Against

A claim that the enzymatic route is greener is credible only if the chemical route it replaces is described fairly. For an ester bond, the classical options are activation of the acid as an acyl chloride or, for sensitive substrates, carbodiimide-mediated (Steglich) coupling with DCC or EDC and a nucleophilic catalyst such as DMAP [80]. A third classical option deserves naming precisely because it resembles the enzymatic reaction most closely. Direct thermal esterification, the Fischer reaction under Brønsted-acid catalysis or the base-catalyzed transesterification used industrially for sugar esters, needs no coupling reagent and, like the enzymatic route, releases only water. It fails on the substrate rather than on the by-product. In the industrial sucrose-ester process, a soap catalyst at 170 to 185 °C under reduced pressure gives the triester, and the yield reported with lithium oleate is about 38%, whereas the lipase route at 40 to 60 °C gives the 6-*O*-monoester [32]. The comparison that matters is therefore not water against dicyclohexylurea, but mild selectivity against forcing conditions. On a polyhydroxylated promoiety, these methods are not regioselective, so the target hydroxyl must first be exposed by protecting the others and the product then freed by deprotection, the protect–couple–deprotect sequence that adds steps, reagents, and waste. For a glycosidic bond, the classical routes (Koenigs–Knorr with glycosyl halides and heavy-metal promoters, or Fischer glycosidation) likewise rely on extensive protecting-group manipulation to control regio- and stereochemistry [30,129]. For a phosphate, direct phosphorylation of a poorly reactive alcohol is difficult and the intermediates are prone to decomposition, so protected phosphoramidite or dibenzyl-phosphate chemistry is used and later removed [130].

**Scheme 6:**
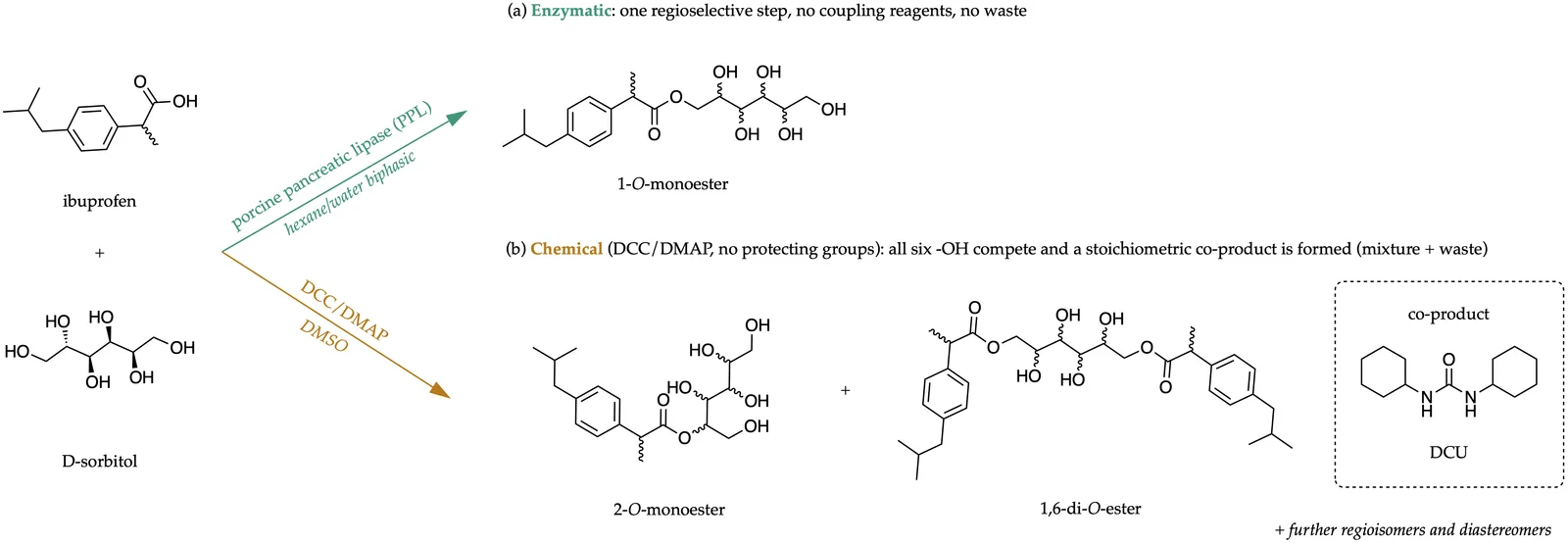
One molecule, two routes: the ibuprofen–sorbitol ester as an unwanted impurity and as a designed prodrug. (**a**) Enzymatically, porcine pancreatic lipase in a hexane/water biphasic medium gives a single regioselective primary-hydroxyl monoester without coupling reagents, releasing only water [16]. (**b**) Chemically, DCC coupling with catalytic DMAP leaves all six sorbitol hydroxyls free to compete: Douša and co-workers obtained a racemic mixture of four isomers, mainly the sorbit-1-yl and sorbit-6-yl 2-(4-isobutylphenyl)propionates, which are the two assigned in the isolated standard, together with stoichiometric dicyclohexylurea (DCU) to be filtered off; there, the ester is a degradation impurity of ibuprofen soft-gelatin capsules, prepared only as a reference standard [131]. Wavy bonds mark centers of undefined configuration: the α-carbon of racemic ibuprofen and the polyol carbons that are esterified. Positions are drawn rather than assigned: C-1 is not distinguished from C-6 in the cited work, and the chemical products are those expected from acylation without protecting groups. Table 5 tabulates the process attributes of the two routes.

Against this benchmark, the enzymatic advantage is specific rather than universal. On a substrate bearing an accessible, non-competing hydroxyl, a lipase or glycosyltransferase creates the same bond in a single regioselective step under mild, near-neutral conditions, without protection or deprotection and often with the biocatalyst recoverable by immobilization; the contrast is made concrete for the ibuprofen–sorbitol ester in Scheme 6 and Table 5. That particular comparison should be read for what it is. Douša and co-workers prepared the ester as an in-house reference standard for an impurity assay of ibuprofen soft-gelatin capsules, not as a synthetic route; the isomer mixture was the analytical object, and no attempt was made to optimize selectivity, solvent, or waste [131]. It therefore illustrates what unselective acylation of a polyol produces, rather than the best that a deliberate chemical synthesis could achieve, for which the fair benchmark remains the protect–couple–deprotect sequence described above. The saving is therefore largest exactly where the chemical protecting-group burden is largest (the polyols and sugars of Section 3.1 and Section 3.2) and smallest where that burden is unavoidable anyway (the ionizable and competing-nucleophile promoieties of Section 3.3, Section 3.4 and Section 3.5), for which chemistry remains the route in use.

**Table 5:** Process attributes of the two routes to the ibuprofen–sorbitol ester drawn in Scheme 6, read as a green-chemistry contrast: the chemical route, which prepared the ester as an impurity reference standard [131], against the enzymatic route, which prepared it as a designed water-soluble prodrug [16].

Green-Relevant Feature	Chemical Route, [131]	Enzymatic Route, [16]
**Coupling/activating reagents**	DCC (1 equiv) with catalytic DMAP (10 mol%)	**None, direct esterification**
**Stoichiometric co-product**	Dicyclohexylurea (solid) to remove	**Water only**
**Reaction solvent**	DMSO; dichloromethane in work-up	Hexane/water biphasic
**Selectivity**	low, the free hydroxyls compete→a mixture of four isomers, mainly the sorbit-1-yl and sorbit-6-yl monoesters	**Regioselective for a primary –OH**
**Downstream burden**	Chromatographic separation of the isomer mixture	One predominant product (1.1 g isolated; the 67% reported is a conversion yield measured by HPLC)
**Catalyst**	Coupling reagent consumed stoichiometrically	**Cheap free porcine pancreatic lipase: catalytic, not immobilized**
**Reaction conditions**	Room temperature, 20 h	Mild, ~35–40 °C, aqueous–organic
**Role of the ester**	An unwanted degradation impurity (a contaminant)	An intended water-soluble prodrug

### 6.4. The Reporting Gap: Green Metrics, and the Outcomes That Would Justify Them

Whether these qualitative advantages translate into a measured environmental benefit is a separate question, and answering it requires the standard toolkit of green metrics: atom economy, the E-factor (mass of waste per mass of product) and its relative process mass intensity (PMI = E-factor + 1), solvent-intensity measures and, at the most complete level, life-cycle assessment (LCA) [132,133]. These metrics were devised precisely to compare chemical and biocatalytic routes on a common basis, and unified toolkits such as CHEM21 make them straightforward to apply [134]. However, across the enzymatic hydrophilizing-prodrug literature surveyed here they are almost never reported: conversion and yield are given, but E-factor, PMI, solvent intensity, and LCA are largely absent (Table 6). The field asserts greenness far more often than it measures it.

Where quantitative comparisons do exist, they caution against assuming that enzymatic automatically means greener. A full LCA of a nucleotide (2′3′-cGAMP) synthesis found the biocatalytic route roughly eighteen-fold lower in global warming potential than the chemical one [135]; a prospective LCA of a Baeyer–Villiger monomer synthesis found no significant climate change advantage for the biocatalytic route [136]; and a critical evaluation of natural-product glycosylation found that chemical routes could show lower E-factors while biocatalytic routes scored better on endpoint impact categories, showing that a single mass-based metric can mislead [30]. The honest conclusion is not that biocatalysis is proven greener but that, for hydrophilizing prodrugs, its sustainability advantage is still essentially unmeasured. Closing that gap with consistent metrics, and the occasional LCA, is the field’s clearest methodological priority and the bridge to the process and economic questions of the preceding subsections; a complete green account would also credit the renewable supply of the promoiety itself, as for xylitol obtained from lignocellulosic hemicellulose [137].

Because that conclusion carries weight, the basis for the count is set out explicitly rather than asserted. The N = 17 studies are the primary enzymatic and chemo-enzymatic syntheses assembled in Table 3, and each is listed individually, with the corresponding entry for every metric, in Supplementary Tables S1 and S2. A study was included if it reported an original experimental synthesis, catalyzed wholly or in part by an isolated enzyme or a whole cell, of a covalent conjugate between a small-molecule drug or bioactive and a hydrophilizing promoiety (a polyol or sugar alcohol, a mono- or disaccharide, or a glycosyl unit), with experimental detail sufficient to identify the reaction medium and the outcome. Reviews, purely computational or analytical papers, syntheses in the lipophilizing direction, conjugation to macromolecular carriers, and the marketed chemically made prodrugs of Table 4 were excluded, the last because they are not enzymatic and are not what the greenness claim is made about. Each paper was then read for nine green-metric quantities (conversion; isolated yield; the isolated mass, from which a yield can be calculated where no yield is stated; the purity of the isolated conjugate and the technique establishing it; the mass balance of the purification step; a stated green or sustainability rationale for the reaction medium; E-factor; process mass intensity or atom economy; and life-cycle assessment) and for three outcome quantities (a measured aqueous solubility for the conjugate; an in vitro permeability surrogate such as Caco-2 or PAMPA; and any in vivo pharmacokinetic measurement). A metric was scored as reported only where a value, or an explicit rationale in the case of the solvent, appears in the paper itself; a mention of the concept in the introduction or the discussion was not scored. This is an audit of a defined and disclosed set rather than a systematic review, and it is presented so that the reader can extend or contest it; the pattern it returns, however, is not marginal. Conversion and isolated yield were scored separately because they are not interchangeable: the difference between them is what the purification consumed, and it is that difference, together with the purity actually reached, on which any E-factor for these syntheses depends.

The gap is recognized inside the field as well as from outside it. The xylitol monoferulate synthesis states that the work ‘does not include a life-cycle assessment (LCA) to evaluate the CO_2_ footprint of this approach compared to classical esterifications’ and that such an analysis ‘would provide valuable insights into the overall sustainability of the process’ [53]; the niclosamide glucosylation concludes that a comparison of the enzymatic and chemical routes ‘would need to be performed, including using the life-cycle assessment framework to quantify the environmental impacts’ [42]. Two of the seventeen studies, in other words, name the missing measurement themselves. The absence is not of awareness but of measurement, and the lower block of Table 6 shows that the same is true of the endpoint: six of the seventeen report no solubility value for the conjugates they made, and none reports an in vivo measurement.

**Table 6:** What the field measures, and what it does not, across the N = 17 primary enzymatic and chemo-enzymatic prodrug syntheses of Table 3; the upper block covers the green metrics and the lower block the outcome metrics. Six of the seventeen operate in aqueous buffer but none reports a solvent metric or presents the choice as a sustainability decision, and they are therefore not counted in that row. Of the six studies reporting an isolated mass, five give the scale needed to turn it into a yield. Scoring criteria are given in the preceding paragraph.

Metric	Studies Reporting It (n of 17)	Share (%)
***Green metrics***		
Conversion reported	10	59%
Isolated yield reported	2	12%
Isolated mass reported (isolated yield calculable)	6	35%
Purity of the isolated conjugate	0	0%
Mass balance of the purification step	0	0%
Green or sustainability rationale stated for the reaction medium	4	24%
E-factor	0	0%
Process mass intensity or atom economy	0	0%
Life-cycle assessment (LCA)	0	0%
***Outcome metrics***		
Aqueous solubility of the conjugate quantified	11	65%
In vitro permeability surrogate (Caco-2/PAMPA)	1	6%
In vivo pharmacokinetic data	0	0%

### 6.5. Downstream Processing and Product Isolation

Downstream processing is a cost that laboratory reports routinely omit and that manufacturing cannot omit. The very feature that makes a hydrophilizing prodrug useful, high polarity, makes it hard to isolate, because it must be separated from an excess of unreacted polyol or sugar that is equally polar, then freed of solvent and crystallized to the required purity. For polyol and sugar conjugates, this separation, not the enzymatic step, frequently governs net yield, purity, and cost, and it is the least-reported part of the whole sequence [138].

The contents of a published procedure set the limit on what can be computed from it. Supplementary Tables S2 and S3 record, study by study, which quantities each paper gives and which it does not; the counts here summarize them. Not one of the seventeen studies measures the chemical purity of the conjugate it isolated, by which we mean the mass fraction of the isolated solid that is the target compound, determined by a quantitative method such as calibrated HPLC against a reference standard, quantitative NMR against an internal standard, or elemental analysis. Structural identity is provided by NMR and mass spectrometry, except in two studies where it rests on the mass alone; identity establishes what the compound is but not what fraction of the weighed solid it accounts for, and only the second of those two measurements enters a mass balance. Every yield below is therefore an upper bound and every E-factor a lower bound, because an impure solid weighs more than the product it contains, so the true product mass is smaller and the true waste ratio larger. Fifteen of the seventeen purify by chromatography and only one of those states how much eluent that took; in that one synthesis, counting it raises the E-factor by about an order of magnitude, and it is left out of the values reported here. Two studies give an isolated yield; six give an isolated mass, and five of those six also give the scale needed to turn it into a percentage.

We computed E-factors for the polyol conjugates of this survey, because a reader who wants to know whether an enzymatic route wastes less material than a chemical one has no number to consult for any of the syntheses surveyed here: the metric is invoked far more often than it is calculated. The calculation was restricted to one promoiety class, the drug– and bioactive–polyol esters of Section 3.1, and the sugar and glycoside conjugates of Section 3.2 are not costed here. Three reasons decided that scope. The polyol conjugates are the largest group in the survey—nine of the seventeen studies against eight for the sugars. They are the class in which the protecting-group-free argument of Section 3.1 is strongest, so a mass-based metric bears most directly on the verdict. Further, they are the only class for which a chemical route to one and the same conjugate is on record, the ibuprofen–sorbitol ester of Scheme 6, which is what makes a like-for-like comparison possible at all. Those nine studies describe ten conjugates, and nine of the ten state enough for a reaction-level E-factor [132] to be reconstructed, that is, one covering the reaction and its work-up but not the chromatography that follows. No paper computes one, so the values below are ours, and Supplementary Table S3 gives each with the inputs behind it. Four assumptions were needed, each for a stated reason: solvent volumes became masses at 20 °C, because only masses can be added; the immobilized biocatalyst counted as an input, because one batch consumes it; the eluent was excluded wherever its volume is missing, because it cannot be estimated from a composition ratio; and the isolated solid was taken as pure, because no paper says otherwise.

The denominator decides whether the number describes a process or an intention: dividing by a mass that was weighed measures what the process did, while dividing by a theoretical or expected mass measures what it would have done had isolation been free. Six of the nine values rest on an expected product and lie between 49 and 135. Of the remaining three, only one rests on a mass that was weighed, and it is 393; the other two, 26 and 852, rest on a product yield from a chromatographic purification whose basis is not specified in the source, and recomputing those two on the reported conversion instead gives 24 and 647. Values resting on different denominators are not comparable with one another, and ranking all nine together would place the syntheses whose isolation is not reported among the greener ones.

The largest single input is always a solvent, and the stage at which it is consumed is set by the reaction medium. A solventless synthesis, in which the polyol serves as both reagent and medium, appears greener than one that adds an organic solvent, and on the reaction alone it is. The comparison reverses once isolation is counted, because a solventless reaction must extract its product out of a large excess of polyol, whereas a reaction in an organic solvent reaches the column with nothing to separate but the solvent itself. One study made both conjugates. They differ in enzyme, in the enantiomer of ibuprofen used, and in the acid-to-polyol ratio, but the enzyme is under 2% of the input mass in each while the solvent term is 86% and 87%, so the difference between the two values is the stage at which solvent is consumed. The ibuprofen–erythritol ester was made in 5 mL of 2-methyl-2-butanol, needed no extraction, and gives an E-factor of 26. The (*S*)-ibuprofen–glycerol ester was made solventless in glycerol, needed three 10 mL toluene washes, and gives 852, the toluene alone being 87% of the mass entering the process [62]. The solvent that the reaction avoids is consumed instead in the work-up, and in greater quantity. The sorbic acid–glycerol ester repeats the pattern: also solventless, its ethyl acetate wash is 68% of the input mass and raises the value from 125 to 393 [65].

A direct comparison of the two routes to one conjugate was attempted and failed. The ibuprofen–sorbitol ester exists by both. The chemical route states every input, the 0.3 g it isolated, and the gradient from which the eluent follows [131]. The enzymatic route states the 1.1 g it isolated but not the scale that produced it, so its inputs cannot be recovered [16]; the missing quantity is on the enzymatic side. Until preparative scales are reported, the comparison that would settle the question cannot be made even for the one conjugate where both routes exist.

An E-factor that cannot be recomputed from the published procedure cannot be verified by a reader. Table 7 therefore sets out rational reporting guidelines for green metrics in biocatalytic prodrug synthesis: the seven quantities that make an E-factor recoverable from a published procedure, the reason each is required, and what its omission costs. No paper in this survey supplies all seven, so none of the values above are more than a bound.

### 6.6. Process Robustness, PAT/QbD, and Residual-Enzyme Control

Manufacturing also demands robustness and control that a proof-of-concept synthesis need not. Batch-to-batch consistency is the object of quality-by-design, and in-line process-analytical technology (PAT) provides the real-time monitoring that underpins it; here, the in-line and benchtop NMR introduced in Section 4.3 is the natural instrument, turning the same spectroscopy that proves regiochemistry into a tool that could govern conversion and selectivity on the plant within a quality-by-design framework [111,139]. A control point specific to biocatalysis is the enzyme itself: immobilized preparations such as Novozym 435 are known to leach protein and to undergo support dissolution under operating conditions, so residual enzyme or its fragments in the isolated active pharmaceutical ingredient is a genuine quality attribute to be monitored and controlled as part of downstream processing [58,138].

## 7. Chemistry vs. Biocatalysis: A Balanced Verdict

The title asks a question, and the evidence assembled here permits a direct answer, provided it is given class by class rather than as a slogan. Applying the three-part test of Section 1.3 to each promoiety class, and reading the outcome from the verdict map of Table 1, the review reaches three distinct conclusions rather than one.

For the neutral polyol, sugar, and glycoside carriers, chemistry is not the bottleneck: for the bond-forming step, the enzyme is often the better tool. These promoieties present the problem biocatalysis solves best (selecting one hydroxyl among several of near-identical reactivity, on a carrier with no competing reactive group), so a lipase or glycosyltransferase assembles the bond in a single regioselective, protecting-group-free step [49,60]. The solubility gains span roughly 4-fold to 5500-fold for the drug conjugates and larger still for the phytosterol esters (Table 3); for this class, the answer to the title is no, with the docetaxel conjugate as the exception Table 1 records, where a pre-formed glycosyl block is coupled chemically to the drug. The two halves of the class are not equally settled, and the difference is one of cofactor economics. A lipase ester needs no cofactor and the acyl donor is the drug itself, so the mass balance of the enzymatic step is the reaction equation. A Leloir glycosyltransferase, by contrast, consumes a sugar nucleotide, and UDP-glucose is expensive enough that the verdict for glycosides rests on the sucrose-synthase and co-immobilized cascade systems of Section 2.2, which keep the nucleotide catalytic rather than stoichiometric at the cost of a second enzyme, and have so far been demonstrated at laboratory scale [45,61]. For the polyol esters, the answer is therefore unqualified; for the sugar esters, it carries the qualification that Table 1 records, since the ketoprofen conjugates start from an acyl donor prepared chemically; and for the glycosides, it is the same answer with a proviso on cofactor supply that scale-up has yet to discharge. The verdict does not yet claim in vivo performance, since the pharmacokinetic translation of Section 5.4 remains largely unmeasured.

For the ionizable phosphates, the amino acid esters, and the PEG carriers, the verdict inverts: chemistry remains necessary. A phosphate delivers among the largest solubility gains and is the strategy of choice for parenteral formulation, but its bond is made by protecting-group phosphorylation, not by an enzyme; the kinases that transfer phosphate are ATP-dependent and, one engineered exception aside, narrow in scope, while the nonspecific acid phosphatases that transfer it from pyrophosphate are half-saturated at acceptor concentrations far above the solubility of a poorly soluble drug (Section 3.5) [90,94,130]. An amino acid promoiety carries a free α-amine that competes during acylation and a stereocenter that basic coupling can racemize; the marketed amino acid ester prodrugs are made chemically, and the enzymatic route, though conceivable, is not yet practical [75,140]. Each PEG chain end carries a single hydroxyl on an otherwise unreactive ether backbone, so there are no competing sites for an enzyme to distinguish, and the drug-side partner in the camptothecin and SN38 conjugates is the hindered tertiary C-20 hydroxyl, so these conjugates are built by chemical activation throughout [77,79]. For these three classes the present answer is yes, chemistry is still required, but the qualifier ‘present’ is deliberate: the one enzymatic attempt on the amino acid ester bond was limited by the same amine competition it would have to overcome (Section 3.3), so the barrier there is one of present capability rather than of principle.

The third conclusion concerns the claim most often used to justify the enzymatic route, that it is greener, and here the verdict is neither yes nor no but not proven. The metrics that would settle it exist and were designed for exactly this comparison, yet they are almost never applied to hydrophilizing-prodrug syntheses, and while a reaction-level E-factor can be reconstructed for the few studies that report their preparative scale in full, a complete one cannot be, because only one study reports the eluent consumed in chromatography and none reports the purity actually reached (Section 6.5); where broader LCA comparisons have been made, the biocatalytic advantage proves real in some cases and absent in others [30,135,136]. All of these, moreover, are analogous systems rather than hydrophilizing prodrugs, which have essentially never been assessed this way. Until the field measures rather than asserts, the sustainability leg of the comparison must be reported as an open question, not a settled advantage.

None of this is an argument against chemistry. Classical bond formation remains the more general tool: it is unaffected by a substrate’s incompatibility with water, it handles the ionizable and the sterically hostile with equal ease, it runs on established plant, and it carries decades of regulatory precedent for the very promoieties (phosphate, succinate, amino acid esters) on which marketed solubilizing prodrugs still rest [10]. Biocatalysis earns its place not by displacing this chemistry everywhere but by being demonstrably better where its particular strength, protecting-group-free regioselection, is exactly what the problem requires. The result is therefore a map rather than a case against one side, and it is provisional: it records where the border between chemistry and biocatalysis runs today, and that border is moving (Section 8).

## 8. Challenges, Open Questions, and the Future of Biocatalysis in Prodrug Chemistry

### 8.1. Current Limitations

The enzymatic route to water-soluble prodrugs has four limits that the present evidence does not remove. The first is substrate scope: the low-water organic solvents that drive esterification toward synthesis dissolve the polar promoiety poorly, so substrate solubility, not product solubility, can limit the rate and the attainable titer. The second is control: the balance between synthesis and hydrolysis must be held by managing water activity (Section 2.4), and enzyme stability can suffer with polar substrates and reactive acyl donors. The third is biological: the kinetics of in vivo activation are difficult to program, and genuine pharmacokinetic data for enzymatically made hydrophilizing prodrugs are almost absent (Section 5.4). The fourth is industrial: scale-up and the downstream separation of a polar prodrug from the equally polar excess of polyol or sugar (Section 6.5) both remain to be addressed. None of the four is disqualifying.

### 8.2. Emerging Frontiers

Against these limits stands a set of advances that are moving quickly, and the most consequential is the engineering of the catalyst itself. Directed evolution already extends the scope of the enzymes central to this review (the promiscuity of a natural-product glycosyltransferase, for instance, was broadened by iterative mutation and screening to glycosylate acceptors it had never encountered [48]), yet the bottleneck of evolution is the size of sequence space, which grows exponentially with the number of positions varied and cannot be searched exhaustively, so that functional variants are rare within it. Machine learning-guided directed evolution addresses that limit: by learning the map from sequence to function from characterized variants, statistical models steer the search toward productive regions of sequence space, compressing many rounds of trial and error into far fewer experiments [141]. Beyond redesigning natural enzymes lies the more radical prospect of building them from scratch: computational, increasingly deep learning-driven de novo design is beginning to create entirely new active sites: artificial luciferases have been built around active sites with no counterpart among natural enzymes [142,143], and the same approach has since been applied to the serine hydrolases, the catalytic class to which the lipases and esterases of this review belong [144].

For the classes where chemistry still dominates, these tools point toward three targets that can be written as specifications rather than as aspirations. The first is a phosphotransferase that works at the acceptor concentration a poorly soluble drug can reach, on a drug that presents a hydroxyl to phosphorylate. The nonspecific acid phosphatases have Michaelis constants for the acceptor of 5.3 mM for glucose and 192 mM for inosine, measured on freely soluble sugars and nucleosides rather than on drugs, and a drug that dissolves at the 0.08 to 0.10 mM of phenytoin therefore presents an acceptor concentration between sixty and two thousand times below them (Section 3.5). The enzyme is not the whole problem: at that concentration, the product titer would be about 0.03 g/L against the 14 to 101 g/L at which these enzymes are operated today, so a practical process would also need the drug presented above its aqueous solubility, by cosolvent or as a slurry. Directed evolution has moved that constant, though not far: two rounds of error-prone PCR on the *Morganella morganii* phosphatase lowered it for inosine from 117 to 43 mM, at the cost of a matching fall in V_max_, and lowered the V_max_ of the competing nucleotidase reaction about sixfold [97]. This is the most distant of the three. The second is an acyltransferase or protease that uses an amino acid ester carrying a free α-amine as its acyl donor without acylating that amine, which is one of the two specifications written by the authors of the one attempt on that bond (Section 3.3). Meeting that specification with the enzymes now available means creating a chemoselectivity that the published surveys of these enzymes do not document. The comparison reported for MsAcT between an amine and a hydroxyl is the opposite of the one required, amines being acylated in preference to phenols in para-aminophenol and vanillylamine, and the variants surveyed to 2022 were selected for properties other than selectivity between those two groups; the comparison that bears on this target, between an amine and a primary aliphatic alcohol, is not documented in that survey [36]. In parallel, multi-enzyme cascades that regenerate their own cofactors (Section 2.2 and Section 2.3), continuous-flow operation, and in-line process analytics are the tools that would turn isolated transformations into whole processes, although no continuous-flow synthesis of a water-soluble prodrug has yet been reported (Section 6.1 and Section 6.6) [50,111,125].

The third target addresses the PEG class by changing the carrier rather than by acting on PEG itself, whose single featureless terminus gives an enzyme nothing to select, and it is the target closest to being attempted, because every part of it already exists separately. The PAS polymer is now produced free and strictly monodisperse by microbial secretion at multi-gram titers [85]; engineered peptide ligases acylate a free N-terminal α-amine in aqueous medium, and omniligase-1 has been run at a 100 g scale on the peptide drug exenatide [88]; and reactive residues, or a sortase recognition motif, can be encoded at defined positions along the chain, which is what sortase- or transglutaminase-mediated attachment of a small-molecule payload requires [85,89]. What has not been shown is the acyl donor, because those ligases have been demonstrated with peptide donors, so a small drug in that role is the step the experiment would have to test. Ligase and sortase are alternative routes to that attachment, and they form different bonds, each under the condition set out in Section 3.4. These results have not been assembled into the step Section 3.4 leaves open, the enzymatic formation of a cleavable bond between a poorly soluble drug and a chain long enough to act as a solubilizing promoiety. The experiment is well-defined and the reagents are commercial. It has three endpoints rather than one: forming the bond enzymatically would answer the question of Section 3.4; the aqueous solubility of the isolated conjugate would establish whether a PAS chain acts as a solubilizing promoiety on a small molecule, which no published PAS study reports; and release of the parent drug would establish that the conjugate is a prodrug, because a solubility gain does not by itself make one (Section 3.2).

### 8.3. Open Questions: Toward a Default Biocatalytic Route, and What It Means for the Future of Chemistry

What, then, would it take for the enzymatic route to become the default rather than the exception for water-soluble prodrugs? Three things, each drawn from the preceding sections. First, a shared quantitative language: until the field reports E-factor, PMI, solvent intensity and, where it matters, life-cycle assessment as routinely as it reports yield, the ‘green’ claim will remain an assertion rather than a demonstration (Section 6.4). Second, enzymes engineered for the classes chemistry still owns, phosphates and amino acids above all, so that the verdict map is redrawn by capability, not merely re-described. Third, pharmacokinetic evidence in vivo, without which solubility gains remain promises (Section 5.4).

The title of this review is posed as a rivalry, chemistry or biocatalysis, but the deeper answer may be that the rivalry is dissolving. For most of its history, biocatalysis borrowed what nature happened to have evolved; today, with directed evolution, machine learning, and de novo design, we are increasingly able to write the catalyst to specification [141,143]. Seen this way, the question ‘do we still need chemistry?’ is not about replacing one discipline with another but about the widening of chemistry itself to include the design of the catalysts that build its bonds. Water-soluble prodrugs are a small and concrete part of that shift, and a well-defined place from which to observe it, because here the enzyme’s characteristic strength, protecting-group-free selectivity, is exactly what the problem rewards. This review therefore offers not a verdict that closes the matter, but a description of a boundary that is still moving.

## Figures and Tables

**Table 7:** Rational reporting guidelines for green metrics in biocatalytic prodrug synthesis: the seven quantities that make an E-factor recoverable from a published procedure.

Quantity to Be Reported	Reason	Consequence of Omission
All input masses; solvent volumes	A volume enters only through its density.	No mass balance can be constructed.
Scale of the preparation, not of the screening	It sets the denominator of both values.	No yield and no E-factor follow.
Isolated mass, in grams	It measures the process as performed.	Only a lower bound can be given.
Purity of that material, and the method	An impure solid overstates the product.	The yield is an upper bound, the E-factor a lower bound.
Work-up solvent volume, if extraction is used	Extraction can exceed all other inputs.	A solventless route appears greener.
Eluent volume, or column and gradient	Chromatography can exceed the reaction.	The E-factor omits what can be its largest term.
Meaning of each percentage	Conversion, yield, and recovery differ.	Values differ by tens of percent.

## Data Availability

The dataset underlying the reporting audit of Section 6.4 is provided in full in Supplementary Tables S1 and S2, which list each of the seventeen primary studies against every criterion scored. No other new data were created or analyzed in this study.

## Supplementary Materials

The following supporting information can be downloaded at: https://www.mdpi.com/article/10.3390/pharmaceutics18091166/s1, Table S1: Reporting audit of the seventeen primary studies of Table 3, scored against six green metrics and three outcome metrics, with the inclusion criteria and the scoring rule of Section 6.4; Table S2: Quality of the isolated conjugates, giving for each study the purity reported, the evidence for the site of attachment, the purification and scale, and the isolated yield calculated here; Table S3: Reaction-level E-factors for the polyol esters of Section 3.1, nine studies describing ten conjugates, with the chemical synthesis of the ibuprofen–sorbitol ester for comparison, giving for each the inputs, the denominator used, and the assumptions.

## Abbreviations

The following abbreviations are used in this manuscript:

Abbreviation	Meaning
ALP	Alkaline phosphatase
ATP	Adenosine 5′-triphosphate
AUC	Area under the plasma concentration–time curve
BCS	Biopharmaceutics Classification System
BPHL	Biphenyl hydrolase-like protein (valacyclovir hydrolase)
BsGT110	Glycosyltransferase 110 from *Bacillus subtilis*
CaLA	*Candida antarctica* lipase A
CaLB	*Candida antarctica* lipase B
CES1, CES2	Human carboxylesterase 1 and 2
CGTase	Cyclodextrin glucanotransferase
CHEM21	Chemical Manufacturing Methods for the 21st Century Pharmaceutical Industries (solvent selection guide)
C_max_	Maximum plasma concentration
COSY	Correlation spectroscopy
DCC	*N*,*N*′-dicyclohexylcarbodiimide
DCS	Developability Classification System
DCU	*N*,*N*′-dicyclohexylurea
DEPT	Distortionless enhancement by polarization transfer
DMAP	4-(dimethylamino)pyridine
DMSO	Dimethyl sulfoxide
DNA	Deoxyribonucleic acid
EDC	1-ethyl-3-(3-dimethylaminopropyl)carbodiimide
E-factor	Environmental factor (mass of waste per mass of product)
F	Oral bioavailability
Fer-Ger	Geraniol ester of ferulic acid
GAA	Ganoderic acid A
GH70	Glycoside hydrolase family 70
GT	Glycosyltransferase
GTF-D	Glucosyltransferase-D (glucansucrase) from *Streptococcus mutans*
HMBC	Heteronuclear multiple-bond correlation
HMQC	Heteronuclear multiple-quantum correlation
HSQC	Heteronuclear single-quantum correlation
LCA	Life-cycle assessment
MsAcT	*Mycobacterium smegmatis* acyltransferase
NAC	Near-attack conformation
NMR	Nuclear magnetic resonance
PAT	Process analytical technology
PDB	Protein Data Bank
PEG	Poly(ethylene glycol)
PEPT1	Intestinal peptide transporter 1 (gene SLC15A1)
PK	Pharmacokinetics
PMI	Process mass intensity
QbD	Quality by design
SARS-CoV-2	Severe acute respiratory syndrome coronavirus 2
SGLT2	Sodium–glucose cotransporter 2
SN38	7-ethyl-10-hydroxycamptothecin
T_max_	Time to maximum plasma concentration
UDP	Uridine 5′-diphosphate
a_w_	Water activity
