# Supplementary material for: Do We Still Need Chemistry for Water-Soluble Prodrugs? What Biocatalysis Already Does, and What It Does Not
Source: Pharmaceutics. 2026 Sep 16;18(9):1166. doi: 10.3390/pharmaceutics18091166 (PMC13610416; doi:10.3390/pharmaceutics18091166)
Supplement: Supplementary file 1 [file pharmaceutics-18-01166-s001.zip › pharmaceutics-4517306-supplementary.pdf]

Supplementary Materials

Do We Still Need Chemistry for Water-Soluble Prodrugs? What Biocatalysis Already Does, and What It Does Not

Table S1. Reporting audit of the seventeen primary studies of Table 3, scored against six green metrics and three outcome metrics. Conversion and isolated yield are reported here in separate columns, since the difference between them is what the purification consumed; the quality of the isolated products is audited in Table S2.

#	Study (first author, year) [ref]: conjugate and corresponding row of Table 3	Biocatalyst	Reaction medium	Conversion	Isolated yield	Green rationale for medium	E-factor	PMI / AE	LCA	Solubility of conjugate measured	Permeability surrogate	In-vivo PK
1	Zappaterra 2022 (Int. J. Mol. Sci. 23, 2026) [60]: ibuprofen–xylitol ester (Table 3, row 1)	CaLB (Novozym 435, immob.)	2-methyl-2-butanol, monophasic	yes (80%)	no	no (solvent chosen on logP and solubility grounds)	no	no	no	yes (≈12× vs native ibuprofen; method not stated)	no	no
2	Zappaterra 2021a (Int. J. Mol. Sci. 22, 3066) [16]: ibuprofen–sorbitol ester (Table 3, row 2)	porcine pancreatic lipase (free)	hexane/water biphasic	yes (67%, labeled "conversion yield")	no; 1.1 g isolated	no	no	no	no	NO	no	no
3	Zappaterra 2023 (Appl. Sci. 13, 9852) [62]: ibuprofen–erythritol ester and (*S*)-ibuprofen–glycerol ester (Table 3, rows 3 and 5)	CaLB (Novozym 435); *Rhizomucor miehei* lipase	2-methyl-2-butanol; and solventless, glycerol as reagent and medium	yes (82 ± 4%; 83 ± 5%)	yes (75%, 50%, 63%)	YES (solventless; glycerol justified on safety and handling)	no	no	no	yes (erythritol ester 463 μg/mL, ≈22×, turbidimetric); glycerol ester not measured	no	no
4	Zappaterra 2025 (BioTech 14, 25) [53]: ferulic acid–xylitol ester (Table 3, row 4)	CaLB	*tert*-amyl alcohol	yes (quantitative at 72 h)	no	YES (t-amyl alcohol named a green solvent)	no	no	no [a]	NO	no	no
5	Zappaterra 2024 (Curr. Res. Biotechnol. 8, 100231) [64]: nine cinnamic acids–erythritol esters (Table 3, row 6)	CaLB (Novozym 435, immob.)	solvent-assisted or solventless	yes (91–95%)	no	no	no	no	no	NO	no	no
6	Zappaterra 2021b (Molecules 26, 5966) [63]: UDCA–glyceric ester (Table 3, row 7)	CaLB (Novozym 435, immob.)	solvent-free, glycerol as reagent and medium; the same study also reports a solvent-assisted variant with 2-methylbutan-2-ol, which is the one costed in Table S3	yes (~30%)	no	YES (solvent-free system)	no	no	no	yes (123 mg/L, ≈6× vs 20 mg/L)	no	no
7	Zappaterra 2020 (Fermentation 6, 96) [65]: sorbic acid–glycerol ester (Table 3, row 8)	CaLB (immob.)	solvent-free	yes (61%, labeled "conversion yield")	no as labeled; 101 mg isolated, which is 60.8% on the reported masses	YES (solvent-free system)	no	no	no	NO	no	no
8	Jahangiri 2018 (Food Chem. 268, 203–209) [67]: bixin → sorbitol ester of norbixin (Table 3, row 9)	CaLB (Novozym 435, immob.)	2-methyl-2-butanol + 20% THF, $a_w \approx 0$	yes (50% of SEN, uHPLC)	no	no (THF added to dissolve the substrates; solvent chosen for yield and enzyme activity)	no	no	no	NO (hydrophilicity anticipated, not measured)	no	no
9	Hu 2022 (Food Chem. 370, 131324) [66]: phytosterol–polyol esters (Table 3, row 10)	*C. rugosa* lipase + *B. subtilis* protease	isooctane (step 1), then pyridine (step 2)	yes (>94%)	no	no	no	no	no	yes (4.6–7.9 mM) [b]	YES (Caco-2)	no

10	Shimoda 2011 (Molecules 16, 6769–6777) [39]: docetaxel–monosaccharide conjugates (Table 3, row 11)	glycosidase / transglycosidase + chemical coupling	aqueous (enzymatic step); DMF/BnBr (chemical steps)	no	yes (35%, 22%, 27% enzymatic; 85–95% chemical steps)	no	no	no	no	yes (≈52×)	no	no
11	Wang 2009 (Bioorg. Med. Chem. 17, 1905–1910) [14]: ketoprofen–saccharide conjugates (Table 3, row 12)	lipase (resolution) + *B. licheniformis* protease	pyridine / *tert*-butanol 1:1	no	no; the 22.8 to 87.4% reported there is determined by HPLC	no	no	no	no	NO ("more hydrophilic" only)	no	no
12	Liang 2016 (Sci. Rep. 6, 21051) [49]: flavonoid α-glucosides (Table 3, row 13)	engineered glucansucrase GTF-D M4	aqueous buffer	no	no	no	no	no	no	yes (genistein ≈4×)	no	no
13	Wu 2021a (Antioxidants 10, 1817) [41]: mangiferin glycosides (Table 3, row 14)	maltogenic amylase (*P. galactosidasius*)	aqueous buffer	no	no; 20.1 and 9.3 mg isolated (14.5%, 5.3% calculated here)	no	no	no	no	yes (≈5500×)	no	no
14	Chang 2021 (Biosci. Biotechnol. Biochem. 85, 687–690) [68]: ganoderic acid A diglucoside (Table 3, row 15)	two *Bacillus* glycosyltransferases (Leloir)	phosphate buffer pH 7.4 + UDP-glucose	no	no; 9.6 mg isolated (23.6% calculated here)	no	no	no	no	yes (≈1024×)	no	no
15	Chang 2022 (Agrochemicals 1, 17–21) [69]: ganoderic acid F glucoside (Table 3, row 16)	*Bacillus* GT BsGT110 (Leloir)	phosphate buffer pH 6.0 + UDP-glucose	no	no; 1.7 mg isolated (13.2% calculated here)	no	no	no	no	yes (≈89×)	no	no
16	Wu 2021b (Int. J. Mol. Sci. 22, 9744) [70]: ganoderic acid G glucosides (Table 3, row 16)	*Bacillus* GTs (Leloir)	buffer, pH screened, + UDP-glucose	no	no; 14.4 and 7.8 mg isolated (55%, 30% calculated here)	no	no	no	no	yes (≈97×)	no	no
17	Lax/Welner 2026 (ACS Omega 11, 8667–8677) [42]: niclosamide *O*-glucoside (Table 3, row 17)	engineered plant glycosyltransferases (Leloir)	phosphate-buffered saline with 20% (v/v) DMSO + UDP-glucose	yes (80% of 200 µM)	no	no	no	no	no [c]	yes (≈100×)	no	no
	**TOTAL reporting the metric (n of 17)**			10 (59%)	2 (12%)	**4 (24%)**	**0 (0%)**	**0 (0%)**	**0 (0%)**	**11 (65%)**	**1 (6%)**	**0 (0%)**

Inclusion criteria. A study was included if it reported an original experimental synthesis, catalyzed wholly or in part by an isolated enzyme or a whole cell, of a covalent conjugate between a small-molecule drug or bioactive and a hydrophilizing promoiety (a polyol or sugar alcohol, a mono- or disaccharide, or a glycosyl unit), with experimental detail sufficient to identify the reaction medium and the outcome.

Exclusion criteria. Reviews; purely computational, structural or analytical papers; syntheses in the lipophilizing direction; conjugation to macromolecular carriers (PEG, proteins, antibodies); and the marketed, chemically made prodrugs discussed in the review (fosphenytoin, valacyclovir, valganciclovir and the PEG conjugates), which are not enzymatic and are not the object of the sustainability claim. The seventeen are the syntheses examined as cases in Sections 3.1 and 3.2, which Table 3 presents as representative rather than as a systematic census of the field. Two further enzymatic glycosylations of poorly soluble bioactives are cited in Section 2.2 and are not among them, the resveratrol α-glucoside of Moon et al. [43] and the α-glucosyl-baicalin of Lambert et al. [44]. Scored on the criteria above they behave like the set: both report a conversion and a solubility gain, of 13.5-fold and of 188- to 320-fold, both establish the site of attachment by NMR, and neither reports an isolated yield, an E-factor, a PMI, a life-cycle assessment, a permeability surrogate or in-vivo pharmacokinetics. One difference is worth recording, because it bears on the strongest of the counts above: Lambert et al. give the two purified glucosides as 86.7% and 83.1% pure, on a basis not specified in the article, whereas no purity value appears in any of the seventeen.

Scoring rule. A metric was scored as reported only where a numerical value appears in the paper, or, for the reaction medium, where the paper states a green or sustainability rationale for the solvent chosen. Invocation of "green chemistry" in an introduction or discussion, or affiliation with a laboratory of green chemistry, was not counted. Six of the seventeen studies (entries 12–17) operate in aqueous buffer, a medium that would rank favorably on any solvent-selection guide, but none of the six reports a solvent metric or presents the medium as a sustainability decision, and

they are therefore scored “no” in the green-rationale column. A study reporting a solubility for any conjugate it made is scored “yes” in the solubility column, which is why entry 3 is scored yes although its glyceryl ester was not measured.

Notes. [a] The authors state that the work “does not include a life cycle assessment (LCA) to evaluate the CO_2 footprint of this approach compared to classical esterifications”. [b] For entry 9 the parent value used to compute a gain (2.8×10^{-6} mM) is taken from earlier literature rather than measured alongside the conjugate. [c] The authors state that a comparison of the enzymatic and chemical routes “would need to be performed, including using the life cycle assessment framework to quantify the environmental impacts”. Entry 15 (Chang 2022, ganoderic acid F) is the one study for which the published record supplies neither a conversion nor an isolated yield. No study in the set reports new *in vivo* pharmacokinetic data.

Table S2. Quality of the isolated conjugates: the purity reported, the evidence for the site of attachment, the purification and scale, and the isolated yield calculated here. Chemical purity means the mass fraction of the isolated solid that is the target compound, measured against a standard by calibrated HPLC, quantitative NMR or elemental analysis; identity by NMR and mass spectrometry does not establish it, and no study reports it. The yields calculated here are molar yields on the limiting reagent, computed from the molecular formula of each paper’s own mass spectrum, and are upper bounds. A percentage reported in this literature may be a conversion, an area ratio or a recovery, so reported and recalculated values are not the same quantity and are not compared. One study reports the volume of eluent consumed in purification; the other sixteen give the mobile phase as a composition ratio only.

#	Study [ref]	Purity of the isolated product	Structural evidence for the site of attachment	Purification, scale and isolated mass	Isolated yield calculated here
1	Zappaterra 2022 (Int. J. Mol. Sci. 23, 2026) [60]	not reported	primary –OH; H-1″ assigned at δ 4.22 and 4.12; xylitol is symmetrical, so the two primary positions are constitutionally equivalent	silica gel column; isolated mass not stated	not calculable
2	Zappaterra 2021a (Int. J. Mol. Sci. 22, 3066) [16]	not reported (NMR and HPTLC-MS run)	primary –OH established; C-1 and C-6 of sorbitol not distinguished	silica gel column; 1.1 g isolated	preparative scale not stated
3	Zappaterra 2023 (Appl. Sci. 13, 9852) [62]	not reported	primary –OH of erythritol; enantiomeric pairs (3a)/(3c) and (3b)/(3d) named explicitly	toluene wash, then silica gel column	stated by the authors as product yields from the column, 75%, 50% and 63%; the basis on which they are calculated is not specified in the article
4	Zappaterra 2025 (BioTech 14, 25) [53]	not reported	primary –OH of xylitol; the diester by-product was isolated and characterized	flash column; isolated mass not stated	not calculable
5	Zappaterra 2024 (Curr. Res. Biotechnol. 8, 100231) [64]	not reported	diastereotopic protons 10′ and 10″, with vicinal and geminal coupling constants	silica gel column, then preparative LC; isolated mass not stated. This is the only study in the set whose preparative chromatography is fully specified, in column volumes and flow rates	not calculable
6	Zappaterra 2021b (Molecules 26, 5966) [63]	not reported (uHPLC run)	the two primary –OH of glycerol are constitutionally equivalent; a 50:50 mixture of epimers is stated and chirality deliberately not drawn	silica gel column; isolated mass not stated	not calculable
7	Zappaterra 2020 (Fermentation 6, 96) [65]	not reported (uHPLC run)	1-monoglyceride pattern: δ 4.21 dd (*J* 11.5, 4.4), 4.12 dd (*J* 11.4, 6.3), 3.57 dd (2H); ^{13}C 66.5, 71.2, 64.1	silica gel column; 101 mg from 100 mg sorbic acid	60.8% on the limiting reagent; the paper separately reports a 61% conversion yield, which it defines by an equation as a ratio of chromatographic peak areas
8	Jahangiri 2018 (Food Chem. 268, 203–209) [67]	not reported (uHPLC run on the isolated residue)	none; no NMR was recorded; the sorbitol position is not addressed. The two peaks reported are *cis*/di-*cis* isomers of the polyene chain, summed for quantification	silica gel column; isolated mass not stated	not calculable
9	Hu 2022 (Food Chem. 370, 131324) [66]	not reported	not established	silica gel column; isolated mass not stated	not calculable
10	Shimoda 2011 (Molecules 16, 6769–6777) [39]	not reported	HMBC: H-1a/C-3″ and H-7/C-1″	silica gel column and preparative HPLC	stated by the authors: 35%, 22%, 27% for the enzymatic steps
11	Wang 2009 (Bioorg. Med. Chem. 17, 1905–1910) [14]	not reported (HRMS given for each compound)	^{13}C shifts locate the primary –OH in every product	silica gel column	stated by the authors: 22.8 to 87.4% across the seven conjugates, determined by HPLC
12	Liang 2016 (Sci. Rep. 6, 21051) [49]	not reported (the SDS-PAGE purity is that of the enzyme)	HMBC: Glc-1/C-7 and Glc-1/C-4′	preparative isolation for structure elucidation only	not calculable; the study reports specific activity and regioselectivity, not a yield
13	Wu 2021a (Antioxidants 10, 1817) [41]	not reported	DEPT, HSQC, HMBC, COSY and NOESY	preparative HPLC; 20.1 and 9.3 mg from 100 mg mangiferin in 100 mL	14.5% and 5.3%. The values reported in that study are 10% and 21%, which it states are obtained by dividing the area of the product

					peak by the area of mangiferin in a control run, a different quantity
14	Chang 2021 (Biosci. Biotechnol. Biochem. 85, 687–690) [68]	not reported	HMBC on C-15 and C-26	preparative HPLC; 9.6 mg from 25 mg GAA in 25 mL	23.6%. The value reported in that study is 27%, which is a different quantity
15	Chang 2022 (Agrochemicals 1, 17–21) [69]	not reported	none; the study states that too little material was isolated for NMR	preparative HPLC; 1.7 mg from 10 mg GAF in 10 mL	13.2%. No yield for this product is reported in that study
16	Wu 2021b (Int. J. Mol. Sci. 22, 9744) [70]	not reported	HMBC and NOESY	preparative HPLC; 14.4 and 7.8 mg from 20 mg GAG in 20 mL	55.2% and 29.9%. The values reported in that study are 74% and 35%, which are different quantities
17	Lax/Welner 2026 (ACS Omega 11, 8667–8677) [42]	99%, which is the purity of the niclosamide *O*-glucoside standard obtained there by custom synthesis; the enzymatic reactions in that study are analytical	not applicable	the conjugate used for the biology was purchased	not calculable

Table S3. Reaction-level E-factors for the polyol esters of Section 3.1, nine studies describing ten conjugates, with the chemical synthesis of the ibuprofen–sorbitol ester (entry 11) for comparison; the sugar and glycoside conjugates are outside its scope, for the reasons given in Section 6.5. For each synthesis the table gives the inputs the paper states, the largest single input and its share of the total mass, the quantities the published procedure does not supply, and the denominator used. The values are ours; no paper in the set reports an E-factor. Studies are identified by first author and year, with the full citation and the manuscript reference number in Table S1. Assumptions: solvent volumes converted to masses at 20 °C; the biocatalyst counted as an input; the eluent excluded wherever its volume is not reported, that is everywhere except entries 6 and 11; the isolated solid assumed pure, since no paper reports a purity.

#	Conjugate and route	Input masses and volumes stated	Largest single input	Quantities not supplied by the published procedure	Denominator used	E	What the number is
1	Zappaterra 2022: ibuprofen–xylitol, enzymatic	ibuprofen 338 mg; xylitol 50 mg; Novozym 435 40 mg; 2M2B 5 mL. Conversion 80%	2M2B, 90%	total isolated mass; volume of the THF wash of the biocatalyst; eluent volume	expected product, 89.5 mg	49	lower bound
1b	the same synthesis, second denominator	as above	2M2B, 90%	as above	the 15 mg weighed out for NMR, assumed to come from this preparation	≤296	upper bound; the reaction-level value lies between 49 and 296, the eluent being excluded throughout
2	Zappaterra 2021a: ibuprofen–sorbitol, enzymatic	none for the preparative run. The only reaction volume in the paper is the 15 mL of the optimization screening. At the optimum concentrations it reports, ibuprofen 60 g/L and sorbitol 20 g/L, sorbitol limits at 1.65 mmol and the theoretical maximum in one such run is 0.61 g; even at the highest sorbitol concentration tested, 40 g/L, the ceiling at the reported 67% conversion is 0.82 g. A single 15 mL run therefore cannot account for 1.1 g of product	not applicable	the scale of the preparation	1.1 g weighed	not applicable	not computable. The isolated mass is known and the inputs are not
3	Zappaterra 2023: ibuprofen–erythritol, enzymatic	erythritol 89.8 mg (147 mM, 5 mL); ibuprofen 3 equiv, 454.9 mg; Lipozyme 435 91.0 mg; 2M2B 5 mL. Conversion 82%, stated yield 75%	2M2B, 86%	isolated mass in grams; eluent volume; whether the stated 75% is an overall yield or a recovery from the column	the stated 75%, whose denominator is not specified in the article, applied to erythritol: 171 mg; or the reported 82% conversion: 187 mg	26 or 24	computed on a reported percentage whose basis is not specified in the article; both readings are given
4	Zappaterra 2023: (*S*)-ibuprofen–glycerol, enzymatic	(*S*)-ibuprofen 40.8 mg (66 mM, 3 mL); glycerol 3 mL; Lipozyme RM IM 16.3 mg; toluene 3 × 10 mL. Conversion 83%, stated yield 63%	toluene, 87%	as for entry 3	the stated 63%: 35.0 mg; or the reported 83% conversion: 46.1 mg	852 or 647	computed on a reported percentage whose basis is not specified in the article. The ratio to entry 3 is thirty-three on the first reading and twenty-seven on the second

5	Zappaterra 2025: ferulic acid–xylitol, enzymatic	ferulic acid 100 mg (0.516 mmol, 258 mM); xylitol 15.7 mg (0.103 mmol, 51.6 mM, the limiting reagent); CaLB 200 mg (100 g/L); t-amyl alcohol 2 mL. These are the paper's own optimized conditions, for which it reports a quantitative conversion of xylitol at 72 h and a productivity of 0.7 mmol L^{-1} h^{-1}, which reproduces that conversion	2M2B, 84%	isolated mass; eluent volume. Three different stoichiometries are labeled "5:1" in that article, so the condition costed here is identified by its masses rather than by its label; the >98 ± 2% reported elsewhere in the article belongs to a 5 mL run with 100 mg of molecular sieves in which xylitol is in fivefold excess, not to the conditions costed here	expected product at quantitative conversion of xylitol, 33.9 mg	56	lower bound
6	Zappaterra 2024: cinnamic acid–erythritol, enzymatic	cinnamic acid 120.0 mg (162 mM, 5 mL); erythritol 33.0 mg (acid/alcohol 3:1); Lipozyme 435 50 mg (10 g L^{-1}); 2M2B 5 mL. Yield >95% at 72 h, from the abstract. The paper defines conversion on the acid; at a 3:1 acid/alcohol ratio, and with no diester detected, a conversion above 33% on the acid is not possible, so the reported yield is taken here on erythritol as the limiting reagent	2M2B, 95%	isolated mass; the mass of molecular sieves in the "high" dehydration condition	expected product, 64.7 mg	64, then 1003 to 1256	the only enzymatic entry whose preparative chromatography is fully specified: 31 column volumes of a 150 × 4.6 mm column per batch, that is 77 mL or 61 to 77 g of eluent. 64 is the reaction alone; adding one batch gives 1003 to 1256, and the 65 mg of product require at least two batches, which give 1942 to 2448
7	Zappaterra 2021b: UDCA–glycerol, enzymatic	UDCA 1.50 g (3.82 mmol); glycerol 550 mmol; 2M2B at 1/6 v/v of glycerol; Novozym 435 100 mg; ethyl acetate 3 × 10 mL	glycerol, 60%	the conversion appears only in the figures; isolated mass; eluent volume	expected product at the 80% of Figure 5, 1.43 g	58	lower bound. Figure 4 gives 70% at the same temperature, which would return 67
8	Zappaterra 2020: sorbic acid–glycerol, enzymatic	sorbic acid 100 mg (0.89 mmol, as the paper states); glycerol 10 mL; immobilized CaLB 40 mg; ethyl acetate 3 × 10 mL. The paper also gives the glycerol as 134 mmol, which is 12.34 g against the 12.61 g that 10 mL at 20 °C weighs; on the molar value the E-factor is 391 instead of 393, and 123 instead of 125 without the ethyl acetate	ethyl acetate, 68%	eluent volume	101 mg weighed	393	computed on a weighed mass, the only enzymatic entry for which that is possible; the eluent is not reported and is excluded
9	Jahangiri 2018: bixin → sorbitol ester of norbixin, enzymatic	bixin 59 mg and sorbitol 27 mg, both at 50 mM; Novozym 435 12 mg; 2M2B/THF 3 mL at 80:20. Yield 50% by HPLC	2M2B, 75%	isolated mass; eluent volume	expected product, 40.4 mg	62	lower bound. Sorbitol limits by a 1% margin, which does not change the value
10	Hu 2022: β-sitosterol–sorbitol adipate, enzymatic, two steps	step 1: β-sitosterol 82.9 mg, divinyl adipate 99.1 mg, *C. rugosa* lipase 250 mg, *n*-hexane 10 mL. step 2: intermediate 113.8 mg, sorbitol 109.3 mg, alkaline protease 1000 mg, pyridine 10 mL. Conversion >94% at each step	pyridine, 54%	volumes of the *n*-butanol extraction and the water wash; both eluent volumes; isolated mass	expected product, 133 mg	135	lower bound. Carrying the first step at 94% into the second gives 138; the second step alone gives 82
11	Douša 2017 (J. Chromatogr. Sci. 55, 790–797): ibuprofen–sorbitol, CHEMICAL	D-sorbitol 910 mg; ibuprofen 1030 mg; DMAP 60 mg; DCC 1030 mg; DMSO 5 mL; dichloromethane 15 mL	dichloromethane, 70%	nothing. The eluent is computable: Method V is 10.0 min at 20 mL min^{-1}, that is 200 mL, or 180 g of acetonitrile and water	0.3 g weighed, a 16.2% isolated yield	94, then 695	measured. 94 is the reaction and work-up; one chromatographic injection gives 695, itself a floor

References

Reference numbers are those of the main manuscript.

14. Wang, H.-Y.; Li, C.; Wang, N.; Li, K.; Feng, X.-W.; He, T.; Yu, X.-Q. Two-step enzymatic selective synthesis of water-soluble ketoprofen–saccharide conjugates in organic media. *Bioorg. Med. Chem.* **2009**, *17*, 1905–1910. https://doi.org/10.1016/j.bmc.2009.01.040

16. Zappaterra, F.; Maldonado Rodriguez, M.E.; Summa, D.; Semeraro, B.; Costa, S.; Tamburini, E. Biocatalytic Approach for Direct Esterification of Ibuprofen with Sorbitol in Biphasic Media. *Int. J. Mol. Sci.* **2021**, *22*, 3066. https://doi.org/10.3390/ijms22063066

39. Shimoda, K.; Kubota, N. Chemo-enzymatic synthesis of ester-linked docetaxel–monosaccharide conjugates as water-soluble prodrugs. *Molecules* **2011**, *16*, 6769–6777. https://doi.org/10.3390/molecules16086769

41. Wu, J.-Y.; Ding, H.-Y.; Wang, T.-Y.; Tsai, Y.-L.; Ting, H.-J.; Chang, T.-S. Improving Aqueous Solubility of Natural Antioxidant Mangiferin through Glycosylation by Maltogenic Amylase from Parageobacillus galactosidasius DSM 18751. *Antioxidants* **2021**, *10*, 1817. https://doi.org/10.3390/antiox10111817
42. Lax, B.M.; Escorial García, M.; Fernandez-Antunez, C.; Bukh, J.; Ramirez, S.; Welner, D.H. Enzymatic Glucosylation Enhances the Solubility of Niclosamide but Abrogates Its Therapeutic Efficacy. *ACS Omega* **2026**, *11*, 8667–8677. https://doi.org/10.1021/acsomega.5c12185
49. Liang, C.; Zhang, Y.; Jia, Y.; Wang, W.; Li, Y.; Lu, S.; Jin, J.-M.; Tang, S.-Y. Engineering a Carbohydrate-processing Transglycosidase into Glycosyltransferase for Natural Product Glycodiversification. *Sci. Rep.* **2016**, *6*, 21051. https://doi.org/10.1038/srep21051
53. Zappaterra, F.; Presini, F.; Meola, D.; Chaibi, C.; Aprile, S.; Lerin, L.A.; Giovannini, P.P. Green biocatalysis of xylitol monoferulate: Candida antarctica lipase B-mediated synthesis and characterization of a novel bifunctional prodrug. *BioTech* **2025**, *14*, 25. https://doi.org/10.3390/biotech14020025
60. Zappaterra, F.; Tupini, C.; Summa, D.; Cristofori, V.; Costa, S.; Trapella, C.; Lampronti, I.; Tamburini, E. Xylitol as a Hydrophilization Moiety for a Biocatalytically Synthesized Ibuprofen Prodrug. *Int. J. Mol. Sci.* **2022**, *23*, 2026. https://doi.org/10.3390/ijms23042026
62. Zappaterra, F.; Presini, F.; Venturi, V.; Lerin, L.A.; Giovannini, P.P.; Costa, S. Biocatalytic Insights for the Synthesis of New Potential Prodrugs: Design of Two Ibuprofen Derivatives. *Appl. Sci.* **2023**, *13*, 9852. https://doi.org/10.3390/app13179852
63. Zappaterra, F.; Costa, S.; Summa, D.; Semeraro, B.; Cristofori, V.; Trapella, C.; Tamburini, E. Glyceric Prodrug of Ursodeoxycholic Acid (UDCA): Novozym 435-Catalyzed Synthesis of UDCA-Monoglyceride. *Molecules* **2021**, *26*, 5966. https://doi.org/10.3390/molecules26195966
64. Zappaterra, F.; Meola, D.; Presini, F.; Aprile, S.; Venturi, V.; Nosengo, C.; De Luca, C.; Catani, M.; Lerin, L.A.; Giovannini, P.P. Differential effect of nine cinnamic acid derivatives on the biocatalytic activity of Candida antarctica lipase type B. *Curr. Res. Biotechnol.* **2024**, *8*, 100231. https://doi.org/10.1016/j.crbiot.2024.100231
65. Zappaterra, F.; Summa, D.; Semeraro, B.; Buzzi, R.; Trapella, C.; Ladero, M.; Costa, S.; Tamburini, E. Enzymatic Esterification as Potential Strategy to Enhance the Sorbic Acid Behavior as Food and Beverage Preservative. *Fermentation* **2020**, *6*, 96. https://doi.org/10.3390/fermentation6040096
66. Hu, Y.-Y.; Ma, C.-G.; Zhou, T.-L.; Bai, G.; Guo, S.-J.; Chen, X.-W. Enzymatic synthesis of hydrophilic phytosterol polyol esters and assessment of their bioaccessibility and uptake using an in vitro digestion/Caco-2 cell model. *Food Chem.* **2022**, *370*, 131324. https://doi.org/10.1016/j.foodchem.2021.131324
67. Jahangiri, A.; Møller, A.H.; Danielsen, M.; Madsen, B.; Joernsgaard, B.; Vaerbak, S.; Adlercreutz, P.; Dalsgaard, T.K. Hydrophilization of bixin by lipase-catalyzed transesterification with sorbitol. *Food Chem.* **2018**, *268*, 203–209. https://doi.org/10.1016/j.foodchem.2018.06.085
68. Chang, T.-S.; Chiang, C.-M.; Wu, J.-Y.; Tsai, Y.-L.; Ting, H.-J. Production of a new triterpenoid disaccharide saponin from sequential glycosylation of ganoderic acid A by 2 Bacillus glycosyltransferases. *Biosci. Biotechnol. Biochem.* **2021**, *85*, 687–690. https://doi.org/10.1093/bbb/zbaa055
69. Chang, T.-S. Glycosylation of ganoderic acid F by Bacillus glycosyltransferase. *Agrochemicals* **2022**, *1*, 17–21. https://doi.org/10.3390/agrochemicals1010003
70. Wu, J.-Y.; Ding, H.-Y.; Wang, T.-Y.; Zhang, Y.-R.; Chang, T.-S. Glycosylation of ganoderic acid G by Bacillus glycosyltransferases. *Int. J. Mol. Sci.* **2021**, *22*, 9744. https://doi.org/10.3390/ijms22189744
131. Douša, M.; Meca, L.; Gibala, P.; Jirman, J.; Tkadlecová, M.; Srbek, J.; Šalandová, J.; Kovalčíková, E.; Břicháč, J. Esterification of Ibuprofen in Soft Gelatin Capsules Formulations – Identification, Synthesis and Liquid Chromatography Separation of the Degradation Products. *J. Chromatogr. Sci.* **2017**, *55*, 790–797. https://doi.org/10.1093/chromsci/bmx036
